# Pro-Apoptotic Organometallic Sn(IV) Bis-Organosilane Benzoate Complexes with Sustained Reduction in Cell Viability Under Resistance-like Conditions in Colorectal Cancer Cells

**DOI:** 10.3390/ijms27157053

**Published:** 2026-08-06

**Authors:** Alberto Galindo-Caballero, Francisco Navas, Ana Belén Griso-Acevedo, Victoria Morales, Ana Sastre-Perona, Raúl Sanz, Rafael A. García-Muñoz

**Affiliations:** 1 Group of Chemical and Environmental Engineering, Rey Juan Carlos University, 28933 Móstoles, Spain; alberto.galindoc@urjc.es (A.G.-C.); francisco.navas@urjc.es (F.N.); victoria.morales@urjc.es (V.M.); raul.sanz@urjc.es (R.S.); 2 Instituto de Investigación de Tecnologías para la Sostenibilidad, Rey Juan Carlos University, 28933 Móstoles, Spain; 3 Laboratory of Translational Research in Maxillofacial Surgery and Head and Neck Cancer, IdiPAZ, 28046 Madrid, Spain; anabgriso98@gmail.com (A.B.G.-A.); amsp84.as@gmail.com (A.S.-P.)

**Keywords:** organotin(IV) complexes, colorectal cancer cells, anticancer agents, repeated treatment, adaptative resistance, apoptosis

## Abstract

This work reports the synthesis, characterization and in vitro evaluation of three organometallic triorganotin(IV) complexes bearing a dicarbamido-bis-organosilane benzoate ligand as potential anticancer agents against colorectal cancer cells. The complexes, Sn(IV)-biSi-1, Sn(IV)-biSi-2 and Sn(IV)-biSi-3, differ in the alkyl groups directly bound to the Sn(IV) center (methyl, n-propyl and isopropyl, respectively). Their structures were confirmed by ^1^H, ^13^C and ^119^Sn NMR spectroscopy, FTIR and ESI-MS, supporting monodentate carboxylate coordination and a solvent-dependent Sn(IV) coordination environment. Biological assays in HCT116 cells showed that the final bis-organosilane complexes were markedly more active than their aminobenzoate intermediates and, under several conditions, more effective than cisplatin. Sn(IV)-biSi-2 and Sn(IV)-biSi-3 reduced cell viability to approximately 30% at 0.5 μM after 5 days and to about 11% and 7%, respectively, at 12.5 μM. In repeated-treatment assays, these complexes maintained antiproliferative activity more efficiently than cisplatin, limiting resistance-like cell recovery. Western blot analysis revealed increased γ-H2AX, p53, cleaved caspase-3 and cleaved PARP, indicating DNA damage-associated apoptotic signaling. Overall, these results identify bis-organosilane triorganotin(IV) benzoates, especially Sn(IV)-biSi-2 and Sn(IV)-biSi-3, as promising metal-based anticancer candidates that sustain antiproliferative activity after repeated exposure in colorectal cancer cells and may contribute to strategies aimed at therapy-resistant colorectal tumors.

## 1. Introduction

For several decades, three Pt(II) complexes—cisplatin, carboplatin and oxaliplatin—have been widely used in the treatment of antitumor malignancies because of their powerful antitumor properties and well-known mechanism of action [1]. These Pt(II) drugs bind to the DNA, causing lesions that lead to the activation of programmed cell death [2]. However, their severe side-effects, including dose-limiting toxicity, nephrotoxicity, neurotoxicity, ototoxicity, and myelosuppression [3], in addition to the acquisition of resistance by tumor cells in long-term treatments [4], has caused an intense and alternative search for new metal-based drugs as antitumor agents to overcome these limitations [5,6,7]. Among these metallodrugs, organotin(IV) organometallic complexes have recently appeared as promising antitumor candidates because of their good results in terms of cytotoxic activity in vitro and in vivo against a wide panel of different types of tumors [8,9,10,11], and are considered one of the fastest developing fields in biomedical and bioinorganic chemistry in the last few years [11]. Moreover, one of the most significant attributes of Sn(IV) compounds is their ability to avoid the development of resistance in tumor cells, and in many cases they also present higher antiproliferative activity and lower induced toxicity and reduced side-effects than cisplatin derivatives [12,13,14,15,16]. It is well known that one of the worst drawbacks of longer chemotherapeutic treatments is the emergence of multi-drug resistance (MDR), a phenomenon in which cells are no longer sensitive to the biological action of the administered drugs [17,18]. Therefore, the use of these organotin(IV) compounds in cancer treatments could result in a promising and innovative strategy to overcome this critical deficiency. These complexes present at least one Sn-C covalent bond [19] and their pharmacological activity is deeply influenced by the chemical structure, the coordination number and the nature of the organic groups directly bound to the metal ion, which directly affect its nucleophilic behavior towards the biological target [11,20]. Thus, they present a central tin(IV) ion covalently bound to one or more organic groups R (R = alkyl, aryl…) and different ligands, L, containing donor atoms, generally -O, -S and -N [8,9,21], which are involved in the transport and delivery of the compound to its biological target [12,22]. Therefore, organotin(IV) compounds present the general formula RSnL [9] and, depending on the number of organic substituents bound to the central ion, they can be classified as RSnL_3_ (monoorganotin), R_2_SnL_2_ (diorganotin), R_3_SnL (triorganotin) or SnL_4_ (tetraorganotin) species [9,23,24]. Furthermore, it has been proposed that the high cytotoxic activity of these organotin(IV) compounds is directly related to the slow hydrolytic decomposition of the Sn–heteroatom bonds, which present a certain stability [8,21,22]. In addition, previous studies presented by Gielsen demonstrated that the organic groups, R, bound to the metal center are more important in the cytotoxic activity of these organotin(IV) compounds than the ligands, L, against different tumors, such as colon and breast carcinomas [25,26].

The mechanism of antitumoral action of the organotin(IV) compounds has been extensively studied, reporting that these agents react to the thiol groups of target proteins [23], with the subsequent DNA binding that activates the apoptotic route via p53 tumor suppression, which leads to final cell death [9,16,23,24,27,28,29]. It has been also noted that triorganotin(IV) complexes are more active than the mono-, di- and tetraorganotin species due to their higher lipophilicity afforded by the three organic groups [30,31], which facilitates their capacity to cross the cell membrane [32,33]. This fact is clearly related to their low water solubility and bioavailability, resulting in a strong hydrophobic character. As has been previously reported, hydrophobic drugs need to be generally administered in high doses to achieve the desired therapeutic effect for their rapid aggregation in blood, generating a rapid withdrawal by macrophages [34]. The low solubility often leads to a rapid binding to plasma proteins, producing the inactivation of the drug and drastically decreasing its biological activity [34]. Additionally, the hydrophobic character notably hinders the evaluation of the biological activity of such compounds, both in vitro and in vivo, since the preparation of samples to carry out this kind of analysis requires aqueous media [11].

For all these reasons, one of the most attractive alternatives used to improve the water solubility in this kind of complex consists of the coordination of the -SnR_3_ fragments with an organic ligand with donor atoms, which also have a crucial role in the geometry and solubility of the metallodrug [14]. In this context, triorganotin(IV) complexes with carboxylate ligands present an increase in water solubility [35,36], in addition to being proven to be the most active in terms of biological activity in human cancer cell lines [13,30,37], even more than other well-known organotin(IV) compounds with thiolato and dithiocarbamato ligands [30]. In these Sn(IV) complexes with carboxylate ligands, the metal can be coordinated with the COO^-^ group in a monodentate form through a single Sn-O bond, presenting the compound with a four-coordination number, or as a bidentate chelate through the two oxygen atoms with a five-coordination number [38]. In the case of a monodentate coordination, there are Sn(IV) carboxylate complexes that present a tetrahedral structure in non-coordinating solvents, such as chloroform, or a trigonal bipyramidal structure in the presence of coordinating solvents (DMSO), adopting a five-coordination number [19,20,39].

The coordination of the ligand, L, with the tin(IV) ion also allows the modification of the molecular structure, affording the introduction of new fragments with different properties that can increase the potential of the pharmacological activity of the metal complex.

Moreover, in recent years, small organic molecules with silicon groups have shown remarkable and promising results both in vitro and in vivo as anticancer agents [40,41]. These organosilicon small molecules exhibit unique properties whereby the replacement of a carbon atom by silicon enhances both the antiproliferative activity and the pharmacological properties of conventional organic compounds [42], while also reducing the toxicity and the undesired side-effects. Because of this, they are currently widely used in medicine and biomedical applications [43,44,45]. Until now, some organosilicon compounds have shown high antitumor activity and an increased uptake by cancer cells in vitro and in vivo, also eliminating the cross-resistance in different tumor cell lines [46,47,48,49,50,51,52].

**Figure 1:**
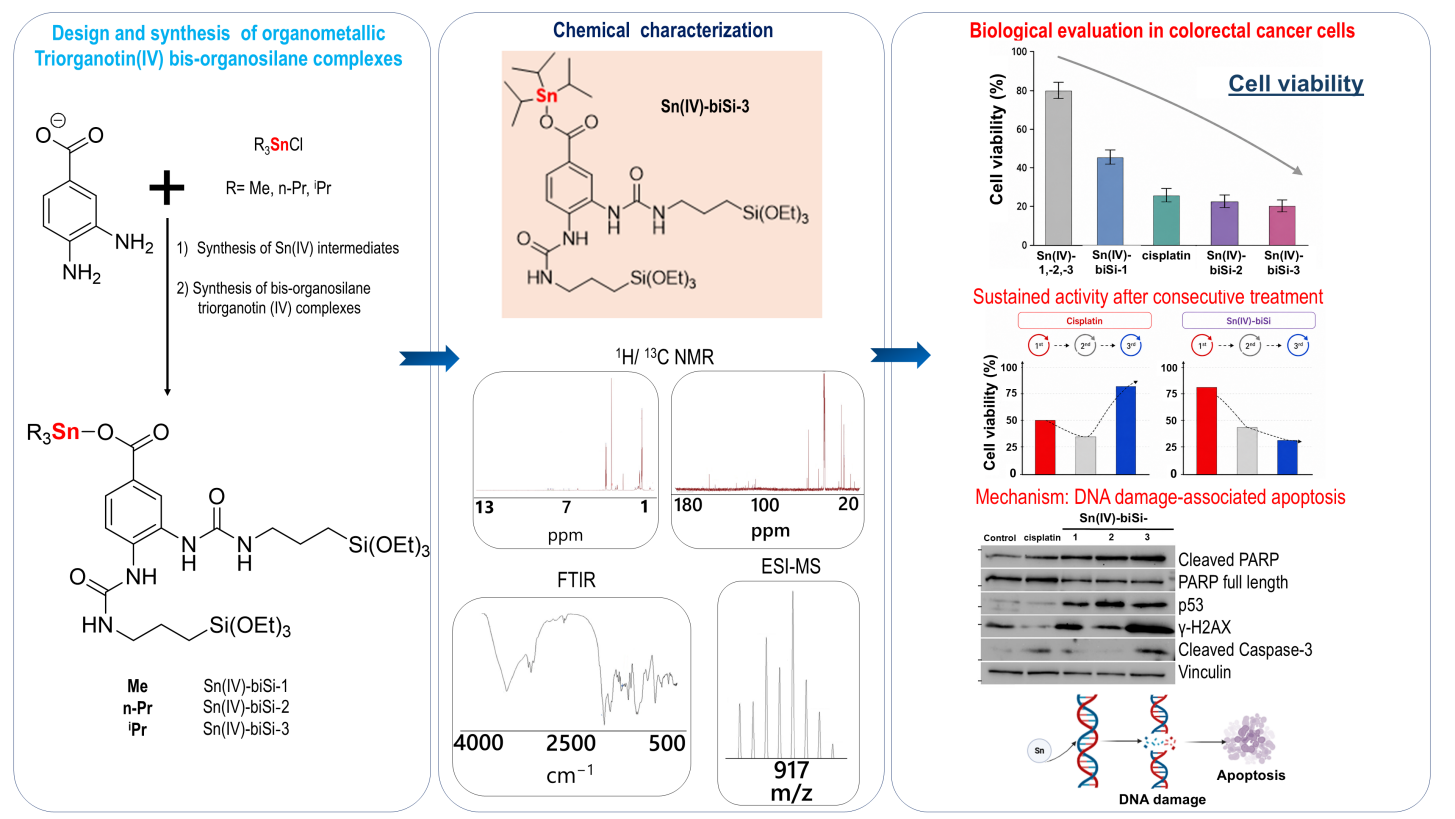
Overview of the synthesis, characterization, and biological evaluation of triorganotin(IV) bis-organosilane complexes. Created with Biorender.com.

In a previous work, we achieved the modification of two well-known Pt(IV) antitumor prodrugs, oxoplatin and iproplatin, by the incorporation of an organic fragment with siloxane terminal groups in the axial positions through the functionalization of the hydroxyl ligands, thereby enabling the generation of Pt(IV) species with improved cytotoxic activity against colorectal tumor cells both in vitro and in vivo [53]. In particular, one of these Pt(IV) compounds with bis-organosilane ligands showed higher activity and selectivity than even cisplatin towards these tumor cells compared with the non-tumorigenic intestinal cells, and additionally demonstrated a marked reduction in tumor growth and lower toxic effects than those produced by cisplatin in a preclinical mice model with colorectal cancer. Therefore, on the basis of these promising results, we designed and synthesized three organometallic triorganotin(IV) complexes with a bis-organosilane benzoate ligand and different alkyl groups directly bound to the metal center (methyl, n-propyl and isopropyl) in order to explore how the organotin alkyl environment and the organosilane-functionalized ligand influence antiproliferative activity in colorectal cancer cells. Rather than claiming a definitive reversal of multi-drug resistance, the present study evaluates whether these complexes maintain activity after repeated exposure cycles, a situation that can reveal adaptive recovery of tumor cells after treatment. Furthermore, the incorporation of the silane fragments was expected to modulate the pharmacological profile of the complexes and potentially enhance their activity relative to cisplatin. In addition, Western blot studies were carried out to gain insight into the molecular mechanism of action of these complexes and to identify the DNA damage- and apoptosis-related signaling pathways involved by evaluating the expression of key proteins associated with apoptosis and cell survival. The overall workflow is summarized in Figure 1.

## 2. Results and Discussion

### 2.1. Synthesis and Characterization of the Organometallic Sn(IV) Complexes

**Scheme 1:**
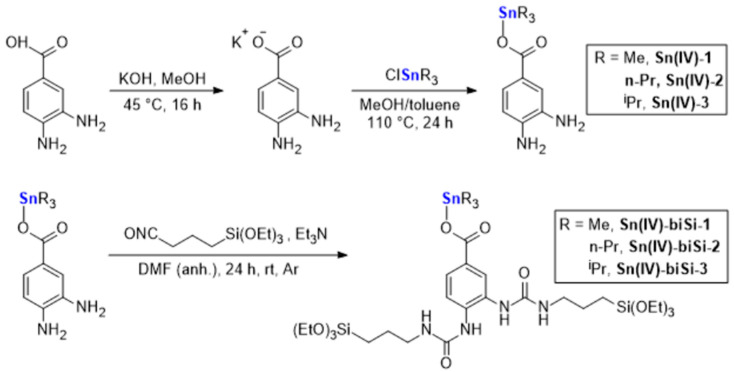
General synthetic procedure to obtain the organometallic intermediates, Sn(IV)-1, -2 and -3, and the final derivatives Sn(IV)-biSi-1, -2 and -3 described in this work.

The synthetic strategy toward the preparation of the desired Sn(IV) compounds incorporating a bis-organosilane ligand started with the preparation of the metal intermediates, Sn(IV)-1 (R = CH_3_), Sn(IV)-2 (R = -CH_2_CH_2_CH_3_) and Sn(IV)-3 (R = -CH(CH_3_)_2_), through the coordination of the diaminobenzoate moiety to each -SnR_3_ fragment (Scheme 1). This process previously required the deprotonation of 3,4-diaminobenzoic acid (DABA) using potassium hydroxide in methanolic solution to afford the corresponding potassium salt (KDAB). This formation was confirmed through the disappearance of the signal corresponding to the -OH group of the carboxylic acid, confirmed by both ^1^H NMR and FTIR. The comparison between the ^1^H NMR spectra of DABA and KDAB clearly showed the disappearance of the strong singlet at 11.7 ppm (Figures S1 and S3, respectively), whereas the FTIR spectra (Figures S2 and S4, respectively) presented the loss of the band corresponding to the *ν*(-OH) at 3440 cm^−1^. The subsequent addition of the corresponding triorganotin(IV) chloride precursor to the KDAB salt in a mixture of methanol and toluene under reflux afforded the formation of the Sn(IV) intermediates, with the removal by filtration of the impurities of KCl formed as a secondary reaction product. The coordination of each -SnR_3_ fragment was confirmed by ^1^H and ^13^C NMR and analysis of the displacement of the signals corresponding to the aliphatic fragments directly bound to the Sn(IV) ion with respect to those of the chloride salt. In all cases, the evaluation of the ^1^H RMN spectra showed a clear shielding of the aliphatic groups bound to the metal center after the coordination with the diaminobenzoate ion.

Comparing the ^1^H RMN spectra between the starting salt SnMe_3_Cl (Figure S6) and the Sn(IV)-1 complex (Figure S7), the singlet corresponding to the protons of the three methyl groups bound to the Sn(IV) ion undergoes clear shielding from 0.54 to 0.43 ppm after the coordination to the diaminobenzoate moiety (Table 1). A lower displacement, from 1.12 to 1.09 corresponding to the -CH_2_- (a) of the propyl fragments directly bound to the Sn(IV) ion, was observed in the spectrum of the Sn(IV)-2 intermediate (Figure S10) with respect to the SnPr_3_Cl salt (Figure S9). Likewise, the signals corresponding to the aliphatic protons of the Sn(IV)-3 complex (Figure S14) also undergo a slight shielding with respect to the salt precursor (Figure S13) Sn^i^Pr_3_Cl (Figure S14). This fact was attributed to the higher inductive effect promoted by the propyl and isopropyl groups over the Sn(IV) ion, causing the hydrogens of the aliphatic chains to be more deshielded, and appearing at higher values of δ compared to the methyl groups. For these complexes, the analysis of the ^13^C NMR spectra did not provide relevant information about the coordination process, because the formation of the R_3_Sn-O bond did not directly affect the electronic distribution of the carboxylate fragment. Thus, there were no significant variations in the δ value corresponding to the signal of the carbon atom after the coordination with the metal ion in comparison to the free ligand at 170 ppm (Figures S3, S7, S10 and S15). Furthermore, FTIR spectra of the metal intermediates were recorded both to confirm the coordination of the ligand with the Sn(IV) ion and to evaluate the mode of this coordination. The band corresponding to the C=O vibration of the carboxylate group of the free ligand appears at 1624 cm^−1^ (Figure S4), moving this signal at values of 1600, 1609 and 1593 cm^−1^ in the spectra of complexes Sn(IV)-1 (Figure S8), Sn(IV)-2 (Figure S11) and Sn(IV)-3 (Figure S15), respectively, with this variation confirming the complexation reaction [54,55]. Moreover, in these complexes the variations in the values (Δν) of the bands corresponding to the OCO asymmetric and symmetric stretching frequencies are used to define the mode of coordination of carboxylate-Sn(IV), because of the coupling presented by the two C=O bonds. In the coordination process, C=O bonds yield electron density to the metal ion, which produces a variation in the C=O bond lengths and, therefore, in the absorption frequency with respect to the free ligand. Defining this variation as Δν = ν_asymm_(OCO) − ν_aymm_(OCO), the coordination can be bridging or chelating if Δν is less than 200 cm^−1^, or monodentate if greater [55,56,57,58]. In the IR spectra of these metal intermediates, this Δν is higher than 200 cm^−1^ (240 cm^−1^ for Sn(IV)-1, 243 cm^−1^ for Sn(IV)-2 and 249 cm^−1^ Sn(IV)), confirming the monodentate coordination with the tin center.

Subsequently, the formation of the bis-organosilane complexes Sn(IV)-biSi-1, Sn(IV)-biSi-2 and Sn(IV)-biSi-3 (Scheme 1) was carried out through the functionalization of the amino groups present in the in the -3 and -4 positions of the aromatic ring. The reaction of both groups against the isocyanate fragment of the organosilylated compound 3-(triethoxysilyl)propyl isocyanate (IPTES) allowed the formation of a carbamide bond in each tail. This process required the presence in the reaction medium of anhydrous triethylamine to enhance the nucleophilic character of the -NH_2_ groups against the electrophilic isocyanate fragment.

The formation of the final Sn(IV) species was also monitored by NMR. The analysis of the ^1^H RMN spectra revealed the disappearance of the two singlets corresponding to the -NH_2_ groups in the metal intermediates (4.86 and 4.43 ppm for Sn(IV)-1 (Figure S7), 4.87 and 4.44 ppm for Sn(IV)-2 (Figure S10) and 4.88 and 4.45 ppm for Sn(IV)-3 (Figure S15)) and the appearance of two triplets associated with the direct coupling between the -NH- groups of the new carbamide fragments (6.85 and 6.47 for Sn(IV)-biSi-1 (Figure S17), 6.15 and 5.79 for Sn(IV)-biSi-2 (Figure S19) and 6.78 and 6.41 for Sn(IV)-biSi-3 (Figure S22)) directly bound to the -CH_2_ group of the aliphatic chain, which appears as a quadruplet at 3.05 ppm in the spectra of Sn(IV)-bisi-1 and -3 and at 2.94 in the spectrum of Sn(IV)-biSi-2. Furthermore, the ^1^H NMR spectra of these final complexes presented the signals from the aliphatic and silylated fragments of the generated tails in the expected range. On the other hand, their ^13^C NMR spectra showed the appearance of two new signals in the carbonyl group area (156.6 and 155.7 ppm for Sn(IV)-biSi-1 (Figure S17), 156.6 and 155.9 ppm for Sn(IV)-biSi-2 (Figure S19) and 163.0 and 156.7 ppm for Sn(IV)-2 (Figure S22), corresponding to the formation of both carbamide groups in each ligand. The IR spectra of these final complexes displayed the appearance of a strong signal at ≈1080 cm^−1,^ corresponding to the Si-(OCH_2_CH_3_) vibration [59] (Figures S18, S20 and S23).

**Figure 2:**
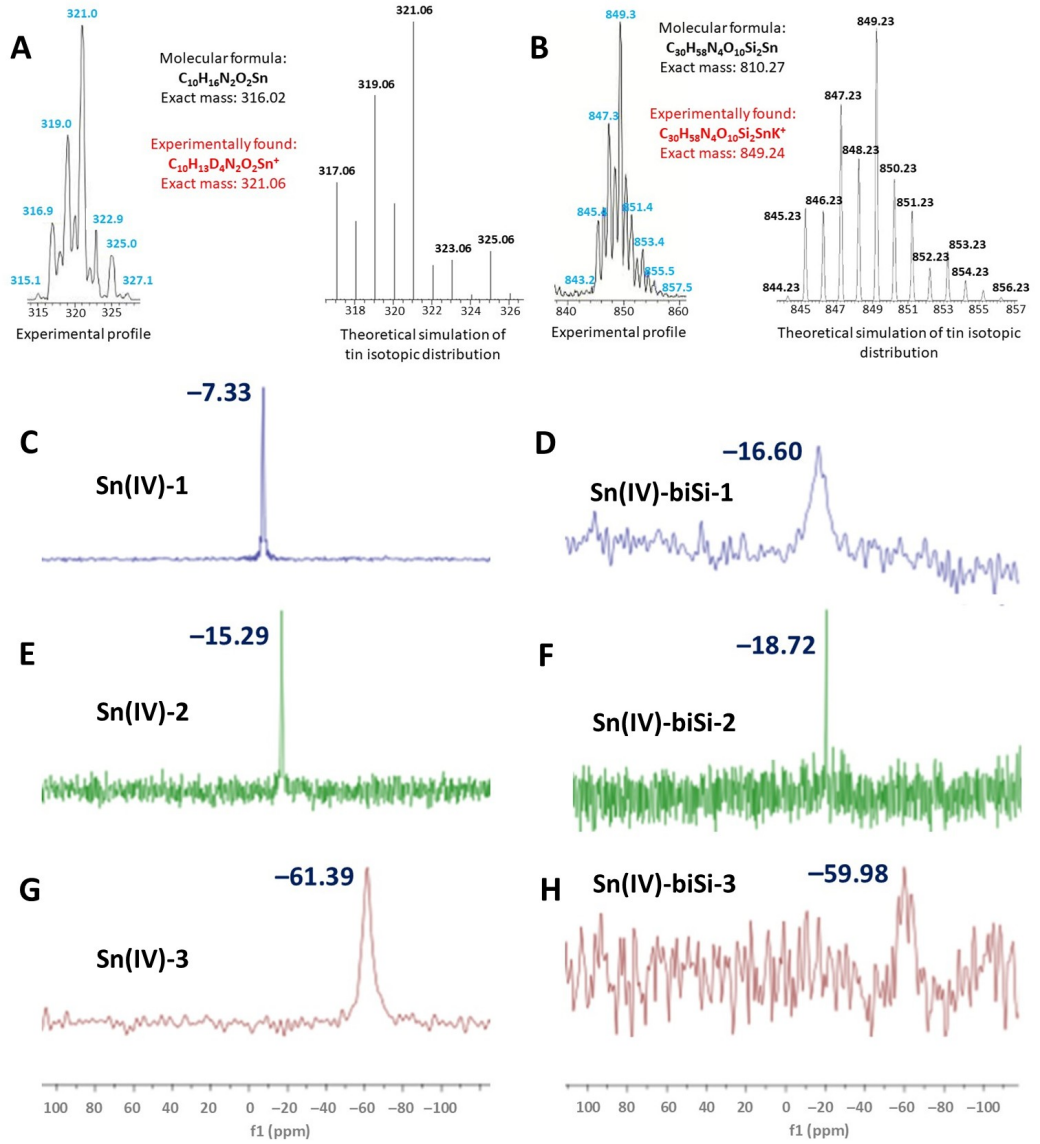
Expanded region of the HRMS (HESI^+^) spectrum of (**A**) Sn(IV)-1 and (**B**) Sn(IV)-biSi-1. Comparison between the experimental isotopic pattern and the theoretical isotopic distribution calculated for the assigned tin-containing ion. ^119^Sn NMR spectra of: (**C**) Sn(IV)-1; (**D**) Sn(IV)-biSi-1; (**E**) Sn(IV)-2; (**F**) Sn(IV)-biSi-2; (**G**) Sn(IV)-3; and (**H**) Sn(IV)-biSi-3. The spectra were vertically offset and normalized for clarity; signal intensities are not directly comparable.

The synthesized compounds were also characterized by mass spectrometry using electrospray ionization both in negative mode for the case of KDAB (Figure S5) and in positive mode for the Sn(IV) complexes (Figure 2A,B for Sn(IV)-1 and Sn(IV)-biSi-1, respectively, and Figures S12, S16, S21 and S24 for Sn(IV)-2, Sn(IV)-3, Sn(IV)-biSi-2 and Sn(IV)-biSi-3, respectively). Each spectrum showed a signal corresponding to the ionized compound and, moreover, for the spectra corresponding to the tin complexes, the tin isotopic distribution pattern in each spectrum unambiguously matches with the theoretical simulation.

Furthermore, the analysis of the ^119^Sn NMR spectra of the Sn(IV) intermediates and the final bis-organosilane species also confirmed their formation. This technique covers a range of more than 600 ppm and, depending on the δ value of each signal in the spectrum, the coordination number around the Sn(IV) ion can be determined [60]. In all the ^119^Sn NMR spectra (Figure 2C–H) only a sharp singlet was observed, indicating the formation of single species. Comparing the spectra of the Sn(IV) intermediates (Figure 2C,E,G), the ^119^Sn NMR resonances of Sn(IV)-2 (−15.29 ppm) and Sn(IV)-3 (−61.39 ppm) are shifted upfield with respect to Sn(IV)-1 (−7.22 ppm), indicating greater shielding of the Sn(IV) nucleus. This behavior can be attributed to the stronger electron-donating character of the three-carbon alkyl substituents compared to the methyl groups in Sn(IV)-1, which increases the electron density around the tin center. In contrast, the markedly higher shielding observed for Sn(IV)-3 relative to Sn(IV)-2 is consistent with the greater electron-releasing ability of the isopropyl groups, resulting from the higher degree of alkyl substitution at the α-carbon directly bonded to the Sn(IV) ion. Furthermore, the increased steric bulk of the isopropyl substituents may lead to subtle changes in the coordination environment around the tin center, which could influence secondary interactions with the carboxylate ligand and contribute to the observed upfield shift of the Sn(IV)-3 resonance [61,62].

The subsequent functionalization of the -NH_2_ groups to reach the formation of the final Sn(IV) complexes (Figure 2D,F,H) hardly produced variations in the δ values with regard to Sn(IV) precursors, because this functionalization took place in distant positions from the metal ion, and did not affect the electronic density of the metal.

The fact that the signals observed in the ^119^Sn NMR spectra of the tin compounds appeared at negative values of δ (^119^Sn) can indicate a higher coordination number around the metal ion, probably 5, also influenced by the presence of coordinating solvents [54], such as DMSO or DMF. Previous studies have demonstrated that most triorganotin carboxylates are present in a solution of non-coordinating solvents—such as chloroform—as monomeric species with a four-coordination number, adopting a quasi-tetrahedral geometry [63,64], with approximate values of δ (^119^Sn) between 100 and 160 ppm. In contrast, the solubilization of these compounds in coordinating solvents produces the variation in a five-coordination number precisely due to the coordination of the solvent molecules to the tin(IV) ion, affording a complex with a trigonal bipyramidal structure (Scheme S1). In this expected structure, the coordinating solvent and the benzoate ligand would be in the axial positions, whereas the aliphatic substituents would occupy the axial positions [55] (Scheme S1).

**Figure 3:**
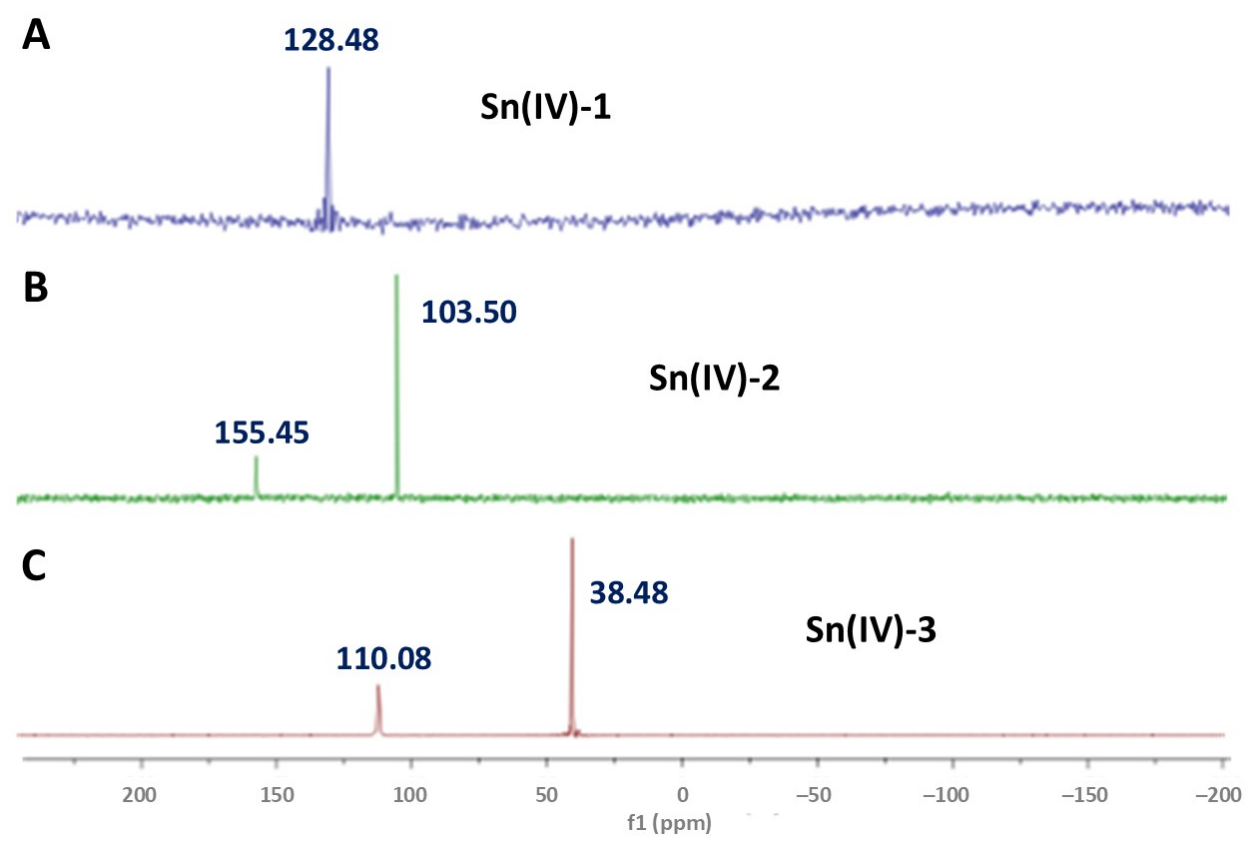
^119^Sn NMR spectra of (**A**) Sn(IV)-1, (**B**) Sn(IV)-2 and (**C**) Sn(IV)-3 in CDCl_3_. The spectra were vertically offset and normalized for clarity; signal intensities are not directly comparable.

Thus, with the aim of determining the influence of the solvent in the coordination around the metal ion in these complexes, ^119^Sn NMR experiments were carried out using the non-coordinating solvent chloroform in its deuterated form, CDCl_3_, comparing the obtained spectra with those previously obtained with DMSO-d_6_. This experiment was performed with the metallic intermediates Sn(IV)-1, -2 and -3, which are completely soluble in chloroform, unlike the final bis-organosilane compounds. The ^119^Sn NMR spectrum for the Sn(IV)-1 compound (Figure 3A) showed only one signal at +128.48 ppm, whereas the spectra of the other two Sn(IV) intermediates bearing three-carbon alkyl substituents displayed two resonances each, at 155.45 and 103.50 ppm for Sn(IV)-2 (Figure 3B) and at 110.08 and 38.48 ppm for Sn(IV)-3 (Figure 3C), consistent with the coexistence of two configurational isomers in each compound. The predominant isomer gives rise to the more intense signal, while the minor isomer accounts for the weaker resonance. The fact that only a single species is observed in both the ^1^H and ^13^C NMR spectra of these intermediates, whereas two signals are detected in the ^119^Sn NMR spectra, clearly indicates that this isomerism can be attributed to the rotation about the Sn–O bond, leading to the formation of two configurational isomers that differ in the relative orientation of the carboxylate ligand environment around the tin center [65,66]. Moreover, the occurrence of rotational isomerism exclusively in the propyl- and isopropyl-substituted Sn(IV) derivatives suggests that the larger three-carbon alkyl groups introduce additional steric and conformational constraints around the tin center. As a result, different rotameric conformations may become energetically distinguishable and interconvert slowly on the NMR timescale, whereas the methyl-substituted analogue remains conformationally less complex and therefore does not exhibit resolvable rotational isomers [67].

Therefore, the values of these positive resonances differ markedly from the negative chemical shifts observed in DMSO-d_6_ (Figure 2C,E,G). These results clearly indicated a variation in the coordination number around the metal center depending on the solvent used, from a four-coordination mode with chloroform to a five-coordination mode caused by the variation with the coordinating solvent DMSO. The molecules of chloroform do not present atoms with free electron pairs that allow the coordination with the tin(IV) ion, meaning that the complex can adopt a quasi-tetrahedral disposition in solution. In the case of the solution of these complexes in DMSO, the cession of the electronic density from the solvent to the metal center, with the formation of a trigonal bipyramidal structure, produces a clear shielding of the tin, shifting this signal at negative values of δ.

The absence of resolved rotational isomers in the ^119^Sn NMR spectra recorded in DMSO can be rationalized by coordination of the solvent molecule with the tin center, leading to the formation of five-coordinate species. The resulting trigonal bipyramidal environment provides a more rigid and symmetric coordination sphere around the Sn(IV) atom, thereby reducing the conformational differences between the rotameric forms and/or promoting their rapid interconversion on the NMR timescale. Consequently, only a single average ^119^Sn resonance is observed in DMSO solution.

The final Sn(IV) bis-organosilane compounds were only soluble in DMSO. Therefore, they are likely to adopt in solution a coordination environment like that proposed for their precursor complexes, possibly involving a trigonal bipyramidal geometry promoted by solvent coordination (Scheme S1).

However, as previously reported, this kind of Sn(IV) compound in the solid state may adopt a different structure from that presented in solution, even forming polymers in which each Sn(IV) ion is bound to two oxygen atoms from different carboxylates [64], presenting a five-coordination number. As this polymeric structure could affect the biological activity of these complexes, we performed a solid-state CP ^119^Sn NMR experiment due to the presence of hydrogen atoms close to the metal. As the formation of this polymer is favored by the presence of alkyl groups with low steric hindrance, we carried out the experiment with the Sn(IV)-biSi-1 compound, which contains methyl groups bound to the Sn(IV). The obtained spectrum (Figure S25) only showed one signal at +112.08 ppm, which unequivocally confirmed the existence of this compound as a monomer in the solid state.

### 2.2. In Vitro Antiproliferative Activity of the Sn(IV) Complexes in Colorectal Cancer Cells

**Figure 4:**
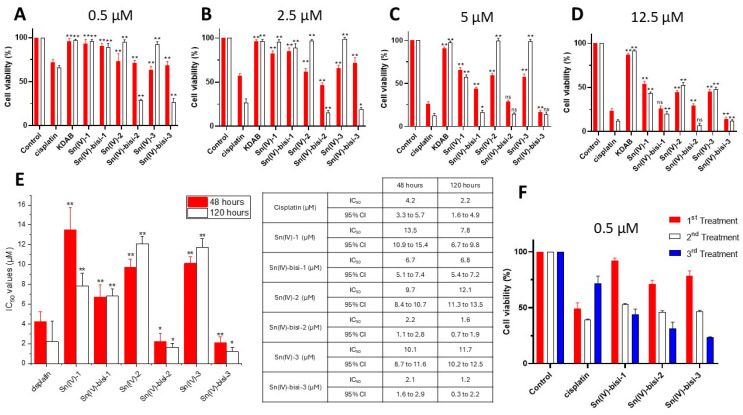
(**A**–**D**) Cell viability (expressed as percentage) of HCT116 cells treated with the selected compounds cisplatin, KDAB, Sn(IV)-1, Sn(IV)-2, Sn(IV)-3, Sn(IV)-biSi-1, Sn(IV)-biSi-2 and Sn(IV)-biSi-3 at concentrations ranging from 0.5 to 12.5 μM for 2 and 5 days. The control represents untreated cells. Cell viability statistical analysis was performed by one-way ANOVA with the test compared with cisplatin at the same concentration, * *p* < 0.001; ** *p* < 0.0001; ns: not significant. (**E**) IC_50_ values of cisplatin, Sn(IV)-1, Sn(IV)-biSi-1, Sn(IV)-2, Sn(IV)-biSi-2, Sn(IV)-3 and Sn(IV)-biSi-3 in HCT116 cells. The bar graph shows the comparative IC_50_ values, and the corresponding numerical values are provided in the table. IC_50_ values were calculated from concentration–response curves as described in the Materials and Methods. Data are presented as mean ± SD from independent experiments. (**F**) Adaptative recovery evaluation expressed as cell viability (%) of HCT116 cells subjected to three consecutive treatment cycles with cisplatin, Sn(IV)-biSi-1, Sn(IV)-biSi-2, and Sn(IV)-biSi-3 at 0.5 μM. Values are means ± SEM of at least 3 independent experiments. IC50 statistical analysis was performed by one-way ANOVA with the test compared with cisplatin at the same concentration, * *p* < 0.01; ** *p* < 0.0001.

With the aim of exploring the pharmacological activity of the Sn(IV) complexes as antitumor agents, we proceeded to monitor their ability to inhibit cell growth. Thus, a panel of concentrations ranging from 0.5 to 12.5 μM was evaluated for each compound, and their antiproliferative effects were monitored after 2 (red bars) and 5 days (white bars) of treatment using the MTT assay, using cisplatin as control (Figure 4A–D). As expected, the KDAB ligand did not affect cell viability at any of the concentrations tested. In contrast, coordination of the Sn(IV)-R_3_ fragment resulted in a substantial enhancement of the growth-inhibitory activity against HCT116 cells.

The cytotoxic potency of the Sn(IV) intermediates was first assessed. Overall, these compounds exhibited only moderate effects on cell viability. The intermediates bearing three-carbon alkyl substituents showed greater biological activity than the Sn(IV)-1 species after 48 h at all concentrations tested. However, following prolonged exposure (5 days), a marked recovery of cell viability was observed, indicating a limited long-term cytotoxic effect. This recovery was less pronounced at the highest concentrations evaluated (5 and 12.5 μM). In contrast, cells treated with the Sn(IV)-1 compound did not exhibit a comparable recovery at concentrations of 5 μM and above. At 5 μM, cell viability decreased from 65% after 2 days to 57% after 5 days, whereas at 12.5 μM viability was reduced from 58% to 43% over the same period. Despite these effects, the cytotoxic potential of the Sn(IV) intermediates remained markedly lower than that of cisplatin, which displayed superior potency under all the experimental conditions evaluated.

A markedly different behavior was observed for the final Sn(IV) bis-organosilane complexes. These compounds displayed substantially greater antiproliferative potency than their corresponding intermediates, and no recovery of cell viability was detected after 5 days of treatment. Furthermore, the complexes containing three-carbon substituents directly bonded to the Sn(IV) center exhibited enhanced growth-inhibitory effects compared with the methyl-substituted analogue. For this compound, cell viability remained around 15% after 5 days of treatment at both 5 and 12.5 μM. Notably, at the highest concentration tested (12.5 μM), its antiproliferative activity was comparable to that of cisplatin. In contrast, the Sn(IV)-biSi-2 and Sn(IV)-biSi-3 complexes already exerted a pronounced antiproliferative effect at the lowest concentration tested (0.5 μM), reducing cell viability to approximately 30% after 5 days of exposure. Their activity increased progressively with concentration, and both compounds displayed very similar dose–response profiles. The strongest effect was observed for the Sn(IV)-biSi-3 complex, which reduced cell viability to approximately 7% at 12.5 μM after 5 days of treatment, whereas the Sn(IV)-biSi-2 analogue reduced viability to about 11% under the same experimental conditions. Importantly, both complexes exhibited greater antiproliferative potency than cisplatin at the lower concentrations tested (0.5 and 2.5 μM) after 5 days of treatment. Moreover, at 5 and 12.5 μM, their activity was comparable to or exceeded that of cisplatin in most cases, highlighting the remarkable efficacy of these Sn(IV) bis-organosilane derivatives.

To further quantify the effects of the compounds on cell viability, IC_50_ values were determined after 48 h and 120 h of treatment (Figure 4E). In agreement with the viability assays, the final Sn(IV) bis-organosilane complexes exhibited the lowest IC_50_ values among all the organotin derivatives. In particular, Sn(IV)-biSi-2 and Sn(IV)-biSi-3 displayed IC_50_ values of 2.2 and 2.1 μM after 48 h, which further decreased to 1.6 and 1.2 μM after 120 h, respectively, both outperforming cisplatin under prolonged exposure (IC_50_ = 2.2 μM at 120 h). In contrast, the Sn(IV)-biSi-1 derivative showed a moderate cytotoxic potency, with IC_50_ values of 6.7–6.8 μM, whereas the corresponding intermediates displayed significantly higher IC_50_ values (6.7–13.5 μM after 48 h). Interestingly, the Sn(IV) intermediates bearing the three-carbon substituents exhibited an increase in IC_50_ values after prolonged incubation, consistent with the recovery of cell viability observed in the dose–response experiments. Overall, the IC_50_ analysis fully supports the results obtained from the MTT assays, confirming that incorporation of the bis-organosilane moiety, particularly in the propyl and isopropyl derivatives, markedly enhances the effect of the Sn(IV) scaffold on cell viability.

Overall, these results clearly demonstrate that the incorporation of bis-organosilane moieties into the Sn(IV) scaffold leads to a substantial enhancement of the effects on cell viability. In particular, the derivatives bearing the three-carbon substituents, propyl and isopropyl, exhibited potencies comparable to, and in some experimental conditions exceeding, those of cisplatin against the HCT116 colorectal cancer cell line.

### 2.3. Repeated-Treatment Assay to Assess Adaptive Resistance-like Recovery In Vitro

Considering the outstanding antiproliferative activity displayed by the Sn(IV) bis-organosilane complexes in HCT116 cells, particularly by the Sn(IV)-biSi-2 and Sn(IV)-biSi-3 derivatives, whose potency was generally comparable to or even greater than that of cisplatin, we next sought to investigate whether these compounds retained activity after repeated treatment cycles. Prolonged exposure to platinum drugs such as cisplatin frequently leads to the development of adaptive cellular responses that diminish therapeutic efficacy and reduce antiproliferative activity [68,69,70]. Accordingly, the following results are discussed as a phenotypic repeated-treatment assay that evaluates adaptive resistance-like recovery after successive exposure cycles.

To evaluate this behavior, HCT116 cells were subjected to three consecutive treatment cycles with the Sn(IV) bis-organosilane complexes, using cisplatin as a reference compound (Figure 4E). Cells were initially exposed to each compound at a concentration of 0.5 μM for 2 days. Following treatment, surviving cells were collected, reseeded, allowed to recover for 2 days, and subsequently subjected to a second treatment under identical conditions. The same procedure was then repeated to complete a third treatment cycle. Overall, this experimental design was intended to evaluate whether HCT116 cells recovered after successive exposures and whether the Sn(IV) complexes preserved their capacity to reduce the number of viable cells across repeated treatment rounds.

During the first treatment cycle, cisplatin exhibited the greatest reduction in viable cell number among all compounds tested, decreasing cell survival to approximately 49%, a significantly greater effect than that observed for any of the Sn(IV) complexes. Among the organotin derivatives, Sn(IV)-biSi-3 was the most active compound during this first cycle, reducing cell survival to approximately 30%, whereas Sn(IV)-biSi-1 and Sn(IV)-biSi-2 showed a more moderate initial effect.

Following the second treatment cycle, cisplatin remained the most active compound. However, the differences between cisplatin and the Sn(IV) complexes became substantially less pronounced. Under these conditions, cisplatin reduced cell survival to approximately 39%, representing only a modest improvement compared with the first treatment cycle. In contrast, the Sn(IV) complexes exhibited an enhanced ability to reduce viable cell number after repeated exposure, with cell survival decreasing to approximately 53% for Sn(IV)-biSi-1 and to approximately 46% for both Sn(IV)-biSi-2 and Sn(IV)-biSi-3 compounds.

The results obtained after the third treatment cycle were particularly noteworthy. Whereas cells exposed to cisplatin at the same concentration displayed a marked increase in cell survival, reaching approximately 71%, the Sn(IV) bis-organosilane complexes continued to reduce viable cell number. Cell survival remained at 44% for Sn(IV)-biSi-1, 23% for Sn(IV)-biSi-2 and 31% for Sn(IV)-biSi-3. These findings suggest that, under the experimental conditions employed, repeated exposure to the Sn(IV) complexes resulted in a sustained reduction in viable cell number compared with cisplatin, particularly for Sn(IV)-biSi-2 and Sn(IV)-biSi-3.

Taken together, these results highlight the potential of these Sn(IV) bis-organosilane derivatives to maintain biological activity during repeated exposure in HCT116 tumor cells. Importantly, these findings should be interpreted within the limitations of the experimental design, as the assay measures changes in viable cell number and does not distinguish between cytotoxic and cytostatic effects. Likewise, although Sn(IV)-biSi-2 and Sn(IV)-biSi-3 consistently outperformed cisplatin under the conditions evaluated, future studies are warranted to further characterize the mechanisms underlying the sustained biological activity of these compounds.

### 2.4. Western Blot Analysis

**Figure 5:**
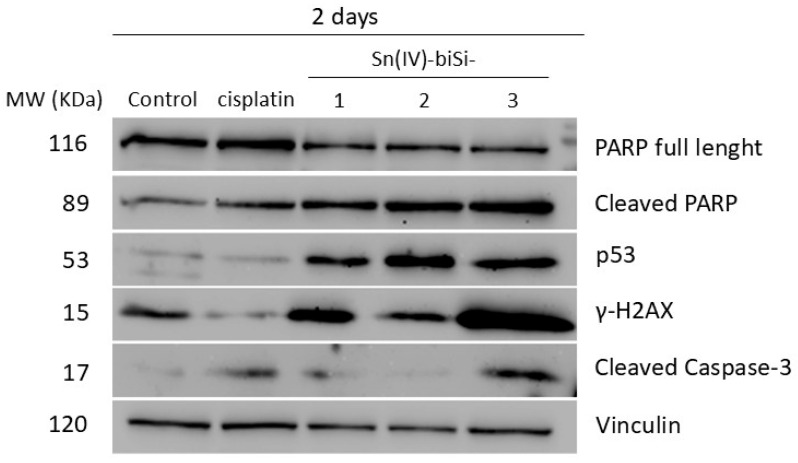
Time-dependent effects of Sn(IV) bis-organosilane treatment on apoptotic and DNA damage-related protein expression in cell lysates after 2 days. Western blot analysis of control, cisplatin-treated, and Sn(IV)-biSi-1-, -2- and -3-treated cells after 48 h. Protein extracts were probed for cleaved PARP and full-length PARP as markers of apoptosis, p53 as a stress response regulator, γ-H2AX as a DNA damage marker, and cleaved caspase-3 as an executioner caspase. Vinculin was used as a loading control.

To gain insight into the molecular mechanisms underlying the cytotoxic potential of the Sn(IV) bis-organosilane complexes, and to evaluate whether their sustained activity after repeated exposure could be associated with stronger engagement of cell-death pathways, the expression of key proteins involved in DNA damage response and apoptotic activation was evaluated by Western blot analysis after 48 h of treatment in HCT116 colorectal cancer cells, using cisplatin as a reference control (Figure 5). The selected biomarkers provide complementary information on different stages of the apoptotic process. PARP (poly(ADP-ribose) polymerase-1) is a DNA repair enzyme with a molecular weight of 116 KDa that is specifically cleaved into fragments of 89 (cleaved PARP) and 24 KDa by activated executioner caspases during apoptosis, making cleaved PARP a well-established hallmark of caspase-dependent cell death [71]. Caspase-3 is the principal executioner caspase responsible for dismantling the cell through the proteolytic cleavage of multiple substrates, including PARP, and its cleaved form (cleaved caspase-3) therefore reflects activation of the apoptotic machinery [72,73]. p53 is a central tumor suppressor that orchestrates the cellular response to genotoxic stress by promoting either DNA repair or apoptosis depending on the extent of damage [74,75]. Finally, γ-H2AX, the phosphorylated form of histone H2AX, is an early and highly sensitive marker of DNA double-strand breaks and activation of the DNA damage response [76].

Compared with cisplatin, treatment with the Sn(IV) bis-organosilane complexes induced a markedly stronger activation of the apoptotic machinery. While cisplatin only elicited a modest increase in cleaved PARP and cleaved caspase-3, Sn(IV)-biSi-1 produced a moderate increase in these apoptotic markers, whereas complexes Sn(IV)-biSi-2 and -3 produced a pronounced accumulation of both proteins, indicating a more efficient activation of the execution phase of caspase-dependent apoptosis. This observation was accompanied by a slight reduction in full-length PARP, and the concomitant appearance of the characteristic 89 kDa PARP cleavage fragment, confirming its proteolytic processing during apoptosis.

Concomitantly, these organotin(IV) compounds triggered a robust activation of the DNA damage response, as demonstrated by the substantial increase in γH2AX phosphorylation, which was already evident for Sn(IV)-biSi-1 and became particularly pronounced for Sn(IV)-biSi-2 and -3. Since γH2AX is an early marker of DNA double-strand breaks, these findings suggest that the organotin compounds induce a more extensive genotoxic stress than cisplatin under the experimental conditions employed.

Importantly, the enhanced DNA damage correlated with a marked accumulation of p53, which was detectable for all three Sn(IV) bis-organosilane complexes but was most prominent following treatment with Sn(IV)-biSi-2 and -3, whereas it was only weakly induced by cisplatin. The simultaneous increase in γH2AX, p53, cleaved caspase-3 and cleaved PARP supports a mechanistic sequence in which DNA damage promotes p53 stabilization, leading to activation of the intrinsic apoptotic program and subsequent caspase-mediated PARP cleavage.

Notably, the molecular profile induced by the Sn(IV) bis-organosilane complexes differs substantially from that observed for cisplatin. Whereas cisplatin produced only limited activation of the DNA damage and apoptotic pathways at the selected time point, Sn(IV)-biSi-1 elicited an intermediate response, while Sn(IV)-biSi-2 and -3 triggered a coordinated response characterized by sustained γH2AX accumulation, p53 stabilization, caspase-3 activation and PARP cleavage. These findings suggest that the superior cytotoxic efficacy of Sn(IV)-biSi-2 and -3 is associated with enhanced DNA damage, which likely contributes to their increased cytotoxicity and the higher levels of apoptosis observed compared with cisplatin. Such enhanced activation of apoptosis may contribute to their sustained activity after repeated treatment cycles. Nevertheless, further studies will be required to fully elucidate the molecular mechanisms underlying the antiproliferative activity of these compounds.

## 3. Materials and Methods

### 3.1. Chemicals

Trimethyltin chloride (98% Sn), tri-*n*-propyltyn chloride (98% Sn), tri-isosopropyltyn chloride (98% Sn) and cisplatin (99% Pt) were purchased from Strem Chemical (Strem Chemicals Inc. (Newburyport, MA, USA)). (3-[4,5-Dimethylthiazole-2-yl]-2,5-diphenyltetrazolium bromide) (MTT), 3,4-diaminobenzoic acid (DABA) (≥97.0%), 3-(triethoxysilyl)propyl isocyanate (IPTES) (95%), toluene, *N*,*N*-Dimethylformamide (DMF, anhydrous, 99.8%), dimethyl sulfoxide (DMSO) and the deuterated solvents dimethyl sulfoxide-d6 (99.8% D) and chloroform-d (99.8% D) were purchased from Sigma-Aldrich (Madrid, Spain). Methanol (99.9%) and potassium hydroxide (85%) were purchased from Honeywell (Charlotte, NC, USA). DMEM medium was purchased from Lonza (Basel, Switzerland). The HCT116 Human colorectal carcinoma cell line was purchased from Cytion (Eppelheim, Germany). RIPA (50 mM Tris-HCL (pH 7.4)), 150 mM NaCl, 1% Triton X-100, 0.5% sodium deoxycholate, 0.1% SDS and 1 mM EDTA in distilled water, supplemented with phosphatase (#04906837001, Roche Diagnostics GmbH, Mannheim, Germany) and protease inhibitors (#HY-K001, MedChemExpress LLC, Monmouth Junction, NJ, USA). Primary antibodies for Western Blot: anti-cleaved caspase-3 (#9664, Cell Signaling Technology, Danvers, MA, USA; 1:1000), anti-p-γH2AX (#2577, Cell Signaling Technology; 1:1000), anti-P53 (#9282, Cell Signaling Technology, 1:1000), anti-Vinculin (#sc-73614, Santa Cruz Biotechnology, Dallas, TX, USA; 1:1000). Secondary antibodies for Western Blot: anti-Rabbit HRP (#A27036, Invitrogen Carlsbad, CA, USA; 1:10.000), anti-Mouse HRP (#AP124P, Sigma-Aldrich, 1:1000–2000). SuperSignal™ West Pico PLUS ECL reagent (ThermoFisher, Waltham, MA, USA).

### 3.2. General Procedures

^1^H and ^13^C NMR analyses were carried out using a Varian Infinity 400 MHz spectrometer equipped with a 9.4 T magnetic field (URJC, Móstoles, Spain) at room temperature. Chemical shifts (δ) are shown in ppm and they were externally referenced to tetramethylsilane (TMS). Minor signals observed in some NMR spectra were assigned to traces of uncoordinated ligand and did not affect the structural characterization of the complexes. ^119^Sn NMR measurements were carried out at 37 °C on a Bruker Avance II/500 MHz spectrometer fitted with a 11.75 T magnetic field (URJC, Móstoles, Spain). Chemical shifts (δ) are shown in ppm and they were externally referenced to tetramethyltin(IV) in DMSO-d_6_ (0 ppm). Room temperature ^1^H–^119^Sn cross-polarization magic-angle spinning (CPMAS) NMR experiments were carried out on a 9.4 T spectrometer equipped with a 4 mm MAS probe. Spectra were acquired using a standard CPMAS pulse sequence at a MAS frequency of 10 kHz. The Larmor frequencies were 399.75 MHz for ^1^H and 149.08 MHz for ^119^Sn. Magnetization transfer from ^1^H to ^119^Sn was achieved using the Hartmann–Hahn condition, employing a contact time of 2.5 ms and recycle delay was set to 2 s.

Mass measurements were performed on an ultra-high-performance liquid chromatography–tandem mass spectrometry (UHPLC-HESI-MS/MS) using a VIP heated electrospray ionization interface (Bruker UHPLC/MSMS EVOQ™ ELITE) with a triple-quadrupole detector (URJC, Móstoles, Spain). The *m/z* values reported in the compound characterization correspond to the molecular or pseudomolecular ions detected by UHPLC-HESI-MS/MS in the full-scan MS acquisition. For clarity, only the ion peak corresponding to each compound is shown in the manuscript; the complete HRMS spectra acquired during the analysis are not displayed. FTIR analyses were performed with the KBr pellet technique on a Mattson Infinity series apparatus in the wavelength range from 4000 to 400 cm^−1^ with a 2 cm^−1^ step size and collecting 64 scans for each analysis (URJC, Móstoles, Spain).

### 3.3. Synthesis of the Organic Ligand and Sn(IV) Complexes

#### Potassium 3,4-Diaminobenzoate (KDAB)

The potassium salt of the 3,4-diamobenzoic acid was synthesized following the next experimental procedure [55]: 1 g of 3,4-diaminobenzoic acid (DABA) (6.57 mmol, 1 equiv.) was dissolved in 30 mL of methanol under continuous stirring at 45 °C. At the same time, a solution of 370 mg of KOH (6.57 mmol, 1 equiv.) in 2 mL of MeOH was prepared and subsequently added to the other solution of DABA, stirring the mixture overnight at 45 °C to reach the formation of the potassium salt. The solution was filtered to eliminate solid impurities, and the filtrate was evaporated under reduced pressure to obtain a red solid. Yield: 85%. %. ^1^H RMN (DMSO-d_6_, 400 MHz): δ 7.11 (d, 1H, J = 1.6 Hz), 7.00 (dd, 1H, J = 7.6 Hz; 1.6 Hz), 6.38 (d, J = 8 Hz), 4.48 (s, 4H). ^13^C NMR (DMSO-d_6_, 400 MHz,): δ 170.7, 138.2, 133.7, 125.8, 120.4, 116.7, 113.3. IR (KBr): 3353 ν(-NH), 1624 ν (C=O), 1575 ν_as_ (COO), 1367 ν_sim_ (COO). HRMS (HESI^−^): *m/z* 151.1 ([M]^−^, calcd for C_7_H_7_N_2_O_2_^−^).

### 3.4. Synthesis of the Tin(IV) Intermediates Complexes

A quantity of 300 mg (1.58 mmol, 1 equiv.) of the potassium salt, KDAB, was dissolved in 5 mL of methanol at 45 °C. To this solution, a solution was added of 1.58 mmol (1 equiv.) of the corresponding triorganotin(IV) chloride species (540.6 mg of trimethyltin chloride and 447.8 mg of tri-*n*-propyltyn chloride or triisopropyltyn chloride) in 15 mL of toluene and the mixture was stirred at reflux for 24 h. The solution was filtered to eliminate solid impurities, and the filtrate was evaporated under reduced pressure to obtain a brown solid.

#### 3.4.1. Trimethyltin(IV) 3,4-Diaminobenzoate—Sn(IV)-1

Yield: 69%. ^1^H NMR (400 MHz, DMSO-d_6_): δ 7.08 (d, 1H, J = 1.2 Hz), 6.99 (dd, 1H, J = 8 Hz; 1.2 Hz), 6.40 (d, J = 8 Hz), 4.86 (s, 2H), 4.43 (s, 2H), 0.43 (s, 9H). ^13^C NMR (400 MHz, DMSO-d_6_): δ 170.9, 133.9, 129.3, 128.6, 120.6, 116.3, 113.1, 1.1. ^119^Sn NMR (500 MHz, DMSO-d_6_): −7.33. IR (KBr, cm^−1^): 3356 ν(-NH); 2927 ν(-CH_3_); 1600 ν(C=O); 1575 ν_as_ (COO); 1335 ν_sim_ (COO). HRMS (HESI^+^): *m/z* 321.06 ([M]^+^·(d_4_), calcd for C_10_H_13_D_4_N_2_O_2_Sn^+^) (This mass spectrometric analysis was performed using the same solutions prepared in deuterated solvents for NMR characterization. The observed deuterated ion resulted from H/D exchange between the complex and the deuterated solvent during sample preparation).

#### 3.4.2. Tri-n-Propylltin(IV) 3,4-Diaminobenzoate—Sn(IV)-2

Yield: 55%. ^1^H NMR (400 MHz, DMSO-d_6_): δ 7.09 (d, 1H, J = 1.6 Hz), 7.01 (dd, 1H, J = 8 Hz; 2 Hz), 6.44 (d, 1H, J = 8 Hz), 4.87 (s, 2H), 4.44 (s, 2H), 1.62 (m, 6H, J = 7.5 Hz), 1.09 (t, 6H, J = 7.5 Hz), 0.93 (t, 9H, J = 7.5 Hz) ^13^C NMR (400 MHz, DMSO-d_6_): δ 171.0, 138.8, 133.8, 124.2, 120.7, 116.7, 113.3, 22.0, 19.5, 19.0. ^119^Sn NMR (DMSO-d_6_): δ −15.29. IR (KBr, cm^−1^): 3367 ν(-NH); 2936, 2887 ν(-CH_2_/-CH_3_); 1630 ν(C=O); 1599 ν_as_ (COO); 1356 ν_sim_ (COO). HRMS (HESI^+^): *m/z* 405.15 ([M]^+^·(d_4_), calcd for C_16_H_25_D_4_N_2_O_2_Sn^+^) (This mass spectrometric analysis was performed using the same solutions prepared in deuterated solvents for NMR characterization. The observed deuterated ion resulted from H/D exchange between the complex and the deuterated solvent during sample preparation).

#### 3.4.3. Triisopropylltin(IV) 3,4-Diaminobenzoate—Sn(IV)-3

Yield: 58%. ^1^H NMR (400 MHz, DMSO-d_6_): δ 7.14 (d, 1H, J = 1.6 Hz), 7.05 (dd, 1H, J = 8 Hz; 2 Hz), 6.43 (d, 1H, J = 8 Hz), 4.88 (s, 2H), 4.45 (s, 2H), 1.58 (m, 1H), 1.28 (s, 18H). ^13^C NMR (400 MHz, DMSO-d_6_): δ 171.7, 139.0, 133.8, 122.8, 120.8, 116.6, 113.1, 67.4, 21.6. ^119^Sn NMR (DMSO-d_6_): δ −61.39. IR (KBr, cm^−1^): 3356 ν(-NH); 2926, 2857 ν(-CH/-CH2/CH_3_); 1609 ν(C=O); 1593 ν_as_ (COO); 1344 ν_sim_ (COO). HRMS (HESI^+^): *m/z* 405.15 ([M]^+^·(d_4_), calcd for C_16_H_25_D_4_N_2_O_2_Sn^+^) (This mass spectrometric analysis was performed using the same solutions prepared in deuterated solvents for NMR characterization. The observed deuterated ion resulted from H/D exchange between the complex and the deuterated solvent during sample preparation).

### 3.5. Synthesis of the Tin(IV) Complexes with Bis-Organosilane Ligands

The synthesis of final Sn(IV) bis-organosilane complexes was performed following the next procedure [53]: 300 mg of Sn(IV)-1 or 379 mg of Sn(IV)-2 and Sn(IV)-3 (0.95 mmol, 1 equiv.), 531 µL (3.81 mmol, 4 equiv.) of anhydrous triethylamine and 475 µL (1.90 mmol, 2 equiv.) of 3-(triethoxysilyl)propyl isocyanate were added to 5 mL of anhydrous DMF under inert atmosphere, and the mixture was stirred at room temperature for 24 h. The final suspension was filtered, and the filtrate was completely removed under reduced pressure. The resulting dark brown oil was washed with petroleum ether to eliminate the excess of isocyanate, stirring vigorously for 2 h.

#### 3.5.1. Trimethyltin(IV) 3,4-Bis(3-(3-(Triethoxysilyl)propyl)ureido)benzoate—Sn(IV)-biSi-1

Yield: 62%. ^1^H NMR (400 MHz, DMSO-d_6_): δ 7.90 (s, 1H), 7.84 (d, 1H, J = 1.6 Hz), 7.75 (dd, 1H, J = 8 Hz; 1.6 Hz), 7.69 (s, 1H), 7.54 (d, 1H, J = 8 Hz), 6.85 (t, 1H, J = 5.2 Hz), 6.47 (t, 1 H, J = 5.2 Hz), 3.74 (m, 12H), 3.05 (q, 4H, J = 6 Hz), 1.49 (m, 4H), 1.14 (m, 18H), 0.55 (m, 9 H), 0.49 (s, 9H). ^13^C NMR (400 MHz, DMSO-d_6_): δ 169.6, 156.6, 155.7, 136.1, 129.0, 125.6, 120.4, 116.0, 113.6, 58.1, 42.5, 24.1, 18.7, 7.7, 1.2. ^119^Sn NMR (DMSO-d_6_): δ −16.60. IR (KBr, cm^−1^): 3360 ν(-NH-); 2927 ν(-CH_3_); 1659 ν(C=O); 1552 ν_as_ (COO); 1343 ν_sim_ (COO); 1080 ν(Si-OCH_2_CH_3_). HRMS (HESI^+^): *m/z* 849.24 ([M + K]^+^, calcd for C_30_H_58_N_4_O_10_Si_2_SnK^+^).

#### 3.5.2. Tri-n-Propyltin(IV) 3,4-Bis(3-(3-(triethoxysilyl)propyl)ureido)benzoate—Sn(IV)-biSi-2

Yield: 51%. ^1^H NMR (400 MHz, DMSO-d_6_): δ 7.96 (s, 1H), 7.73 (d, 1H, J = 1.6 Hz), 7.49 (s, 1H), 7.39 (dd, 1H, J = 8 Hz; 2 Hz), 6.63 (d, 1H, J = 8 Hz), 6.15 (t, 1H, J = 5.2 Hz), 5.16 (t, 1 H, J = 5.2 Hz), 3.75 (m, 12H), 2.94 (q, 4H, J = 6 Hz), 1.65 (t, 6H, J = 7.6 Hz), 1.44 (m, 4H), 1.15–1.11 (m, 24H), 0.94 (t, 9H, J = 7.6 Hz), 0.54 (m, 4 H). ^13^C NMR (DMSO-d_6_): δ 158.4, 156.6, 155.9, 128.9, 126.4, 125.4, 120.3, 115.8, 113.6, 58.1, 42.5, 23.6, 23.5, 19.1, 18.8, 15.0, 7.7. ^119^Sn NMR (DMSO-d_6_): −18.72. IR (KBr, cm^−1^): 3373 ν(-NH-); 2957, 2936 ν(-CH_2_/-CH_3_); 1672 ν(C=O); 1543 ν_as_ (COO); 1355 ν_sim_ (COO); 1082 ν(Si-OCH_2_CH_3_). HRMS (HESI^+^): *m/z* 895.37 ([M + H]^+^, calcd for C_36_H_71_N_4_O_10_Si_2_Sn^+^).

#### 3.5.3. Triisopropyltin(IV) 3,4-Bis(3-(3-(triethoxysilyl)propyl)ureido)benzoate—Sn(IV)-biSi-3

Yield: 54%. ^1^H NMR (400 MHz, DMSO-d_6_): δ 7.95 (s, 1H), 7.87 (d, 1H, J = 1.6 Hz), 7.73 (d, 1H, J = 8 Hz), 7.70 (s, 1H), 7.57 (dd, 1H, J = 8.4 Hz; 1.6 Hz), 6.78 (t, 1H, J = 5.2 Hz), 6.41 (t, 1 H, J = 5.2 Hz), 3.74 (q, 12H, J = 6.8 Hz), 3.05 (q, 4H, J = 6 Hz), 1.62 (m, 1H), 1.49 (m, 4H), 1.32 (d, 18H, J = 7.2 Hz), 1.14 (m, 18H), 0.57 (m, 4 H). ^13^C NMR (DMSO-d_6_): δ 169.8, 163.0, 156.7, 133.9, 124.2, 120.9, 116.4, 114.6, 113.3, 56.5, 46.0, 21.6, 18.9, 18.6, 11.6, 7.6. ^119^Sn NMR (DMSO-d_6_): −59.98. IR (KBr, cm^−1^): 3354 ν(-NH); 2945, 2929 ν(-CH/-CH2/CH_3_); 1659 ν(OCONH); 1556 ν_as_ (COO); 1345 ν_sim_ (COO); 1080 ν(Si-OCH_2_CH_3_). HRMS (HESI^+^): *m/z* 917.36 ([M + Na]^+^, calcd for C_36_H_70_N_4_O_10_Si_2_SnNa^+^).

### 3.6. Cell Culture

Human colorectal carcinoma HCT116 cells were obtained from ATCC and maintained in Dulbecco’s Modified Eagle’s Medium (DMEM) supplemented with 10% fetal bovine serum (FBS) at 37 °C in a humidified incubator under an atmosphere of 5% CO_2_. Cells were seeded at an initial confluence of 30% and allowed to adhere prior to treatment. The indicated complexes were evaluated at concentrations ranging from 0.5 to 12.5 μM, and cells were incubated for either 2 or 5 days, as specified. Stock solutions of the Sn(IV) complexes were freshly prepared in DMSO, whereas cisplatin and KDAB were dissolved directly in Milli-Q H_2_O. All compounds were subsequently diluted in DMEM to the desired working concentrations immediately before use. Vehicle controls containing the same final DMSO concentration were included in all biological assays, and the final DMSO percentage was below 0.1% *v*/*v*, to exclude solvent-related effects on cell viability.

### 3.7. IC50 Assay

HCT116 cells were seeded in 96-well plates at a density of 5 × 10^3^ cells/well and allowed to attach for 48 h at 37 °C in a humidified 5% CO_2_ atmosphere. The medium was then replaced with fresh medium containing the Sn(IV) test compounds or cisplatin over a range of serial concentrations (2.5–20 µM), and control wells. Cell viability was assessed by the MTT assay after 48 and 120 h of exposure. IC50 values for cisplatin, Sn(IV)-1, Sn(IV)-biSi-1, Sn(IV)-2, Sn(IV)-biSi-2, Sn(IV)-3 and Sn(IV)-biSi-3 on the tumor HCT116 cell line were calculated from concentration–response curves by nonlinear regression using GraphPad Prism (version 8.01.1, GraphPad Software, San Diego, CA, USA), applying the four-parameter logistic (4PL) equation: Y = Bottom + (Top − Bottom)/(1 + 10^((LogIC50−X)×HillSlope)^), where X is the logarithm of the compound concentration, Y is the percentage of cell viability, and Top, Bottom, and HillSlope were fitted as free parameters. Experiments were performed independently twice, with each condition tested in duplicate.

### 3.8. Cell Viability by MTT Assay

The MTT assay relies on a colorimetric reaction based on the capacity of living cells to reduce MTT to formazan and change the color to purple, which is easily measured by colorimetry. This essay estimates the cellular viability after the indicated treatments. HCT116 cells were treated with MTT (1:10 in culture medium) and incubated at 37 °C for 3 h. After removal of the medium, the formazan was resuspended in DMSO, transferred to p96 plates, and analyzed by a SpectraFLUOR (Tecan) at 542 nm. In all cases, viability was measured in duplicate of each point in three independent experiments.

### 3.9. Repeated-Treatment Assay

HCT116 cells were subjected to three consecutive treatment cycles with either Sn(IV) bis-organosilane complexes (Sn(IV)-biSi-1, -2 and -3) or cisplatin in a repeated-exposure assay. Cells were seeded in 6-well plates at a density of 25,000 cells per well and allowed to adhere for 48 h. They were then exposed to the corresponding compound at a fixed concentration of 0.5 µM for 48 h (first treatment). After this period, the cells were harvested, counted, and re-seeded at the initial density. Following an additional 48 h incubation period, a second identical treatment was administered. The procedure was repeated once more to complete a third treatment cycle, resulting in three consecutive drug administrations to the same cell population. Cell viability was assessed after each treatment cycle using the MTT assay and expressed relative to untreated control cells cultured in parallel. This assay was used to evaluate maintenance of the effects on cell viability and the recovery of cells after repeated exposure. All experiments were performed in 9 independent biological replicates.

### 3.10. Western Blot

For Western blot analysis, cells were seeded at a density of 50,000 cells per well in 6-well plates. After 24 h, cells were treated with each compound (cisplatin, Sn(IV)-biSi-1, -2 and -3) at a final concentration of 5 µM. Following treatment, the culture supernatant was collected in Falcon tubes kept on ice. Wells were gently washed with PBS 1X and the wash pooled with the corresponding supernatant. The pooled suspension was centrifuged at 450× *g* for 3 min at 4 °C. The supernatant was discarded, and the cell pellet was resuspended in PBS and centrifuged again under the same conditions to wash the detached cell fraction. The remaining adherent cells were lysed with ice-cold RIPA buffer supplemented with protease and phosphatase inhibitors and were thoroughly scraped to ensure complete lysis. After supernatant washing, the PBS was carefully aspirated from the detached-cell pellet, which was then resuspended in the corresponding RIPA lysate recovered from each well. The combined lysates were transferred to microcentrifuge tubes, vortexed, incubated on ice for 10 min, vortexed again, and centrifuged at 12,000× *g* for 10 min at 4 °C. The clarified supernatants were transferred to fresh labeled microcentrifuge tubes, taking care not to disturb the pellet, and protein levels quantified by performing Bradford protein quantification assay (#5000006, Bio-Rad Laboratories, Hercules, CA, USA). Final working samples were prepared in 1X Laemmli Loading Buffer (LB) and SDS-PAGE Western blotting was performed using 20–30 μg of total protein per well in 12% polyacrylamide gels. After gels were transferred to nitrocellulose membranes, these were stained with 0.1% Ponceau in 5% acetic acid and blocked in 5% skimmed milk powder in TBST (1X TBS; 0.05% Tween) for 1 h at RT. Primary antibodies were diluted in either 5% skimmed milk powder or BSA in TBST and incubated overnight at 4 °C on a shaker. Primary antibodies were retrieved, and membranes were washed 3x with TBST before incubating in secondary HRP-conjugated antibodies for 1 h at RT. Membranes were then again washed and incubated for 1 min in chemiluminescent substrate ECL imaging with an UVITEC ALLIANCE imager (UVITEC, Ltd., Cambridge, UK).

## 4. Conclusions

This study introduces a new family of organometallic Sn(IV) bis-organosilane benzoate complexes as molecular candidates for colorectal cancer research. The work demonstrates that combining a triorganotin(IV) pharmacophore with a dicarbamido-bis-organosilane ligand provides a chemically well-defined platform whose biological response is strongly influenced by the organic substituents bound to the tin center. In this context, the *n*-propyl and isopropyl derivatives, Sn(IV)-biSi-2 and Sn(IV)-biSi-3, emerged as the most effective compounds in HCT116 colorectal cancer cells.

Beyond their initial effect on cell viability, these complexes retained a stronger capacity than cisplatin to reduce the number of viable cells after repeated exposure cycles. This result is particularly relevant in the context of adaptive recovery after successive treatments. Mechanistically, the most active derivatives promoted molecular events consistent with DNA damage-associated apoptotic signaling, including activation of p53-related responses, caspase-3 cleavage and PARP processing. Therefore, the present results support the potential of Sn(IV) bis-organosilane benzoates as pro-apoptotic metal-based compounds worthy of further investigation in colorectal cancer models.

## Figures and Tables

**Table 1:** Chemical shift (δ) in ppm of the aliphatic protons of the chloride Sn(IV) salts and the Sn(IV) precursors.

Complexes	*δ* (ppm) -CH-	*δ* (ppm) -CH_2_ (a)	*δ* (ppm) -CH_2_- (b)	*δ* (ppm) -(CH_3_)
ClSnMe_3_	-	-	-	0.54
Sn(IV)-1	-	-	-	0.43
ClSn(nPr)_3_	-	1.12	1.66	0.94
Sn(IV)-2	-	1.09	1.62	0.93
ClSn(^i^Pr)_3_	1.62	-	-	1.28
Sn(IV)-3	1.58	-	-	1.28

## Data Availability

The original contributions presented in this study are included in the article/Supplementary Materials. Further inquiries can be directed to the corresponding author.

## Supplementary Materials

The following supporting information can be downloaded at: https://www.mdpi.com/article/10.3390/ijms27157053/s1.
