# Supplementary material for: Pro-Apoptotic Organometallic Sn(IV) Bis-Organosilane Benzoate Complexes with Sustained Reduction in Cell Viability Under Resistance-like Conditions in Colorectal Cancer Cells
Source: Int J Mol Sci. 2026 Aug 6;27(15):7053. doi: 10.3390/ijms27157053 (PMC13466905; doi:10.3390/ijms27157053)
Supplement: Supplementary file 1 [file ijms-27-07053-s001.zip › ijms-4477155-supplementary.pdf]

SUPPORTING INFORMATION

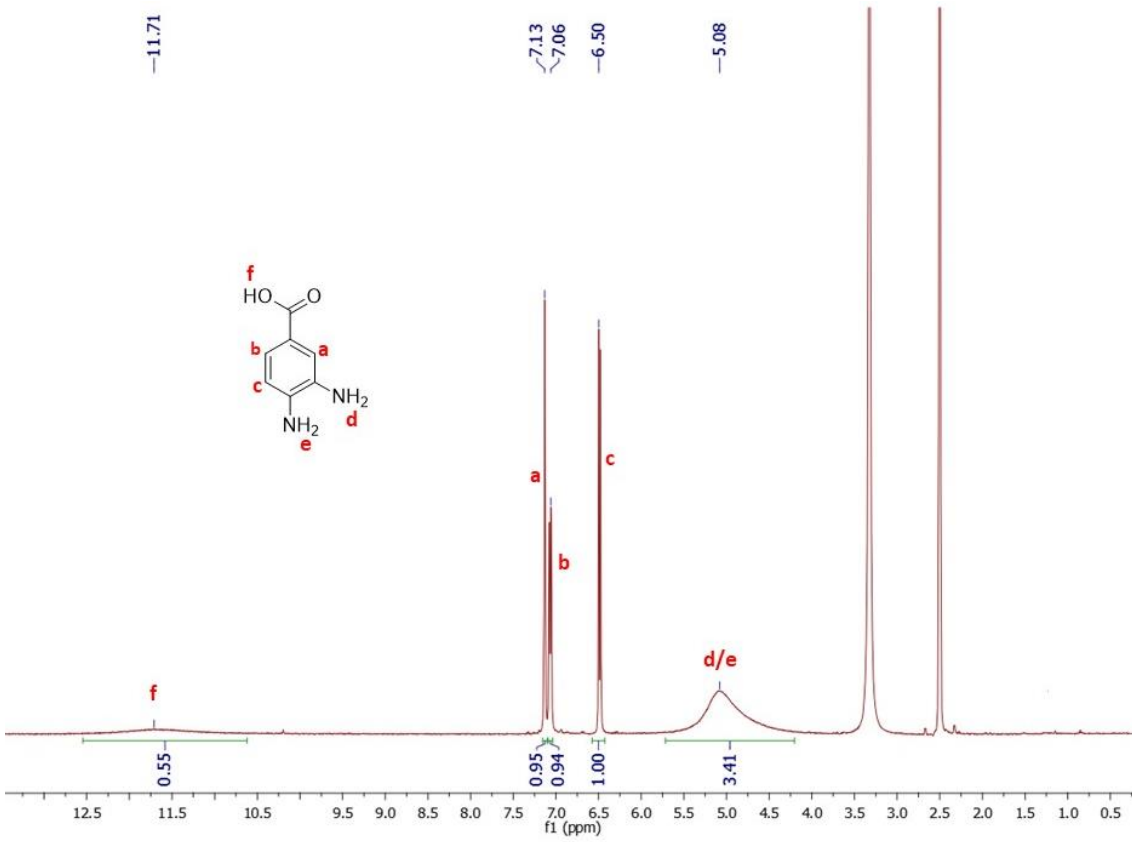

Figure S1. ^{1}H NMR spectra of 3,4-diaminobenzoic acid (DABA) in DMSO-d_6.

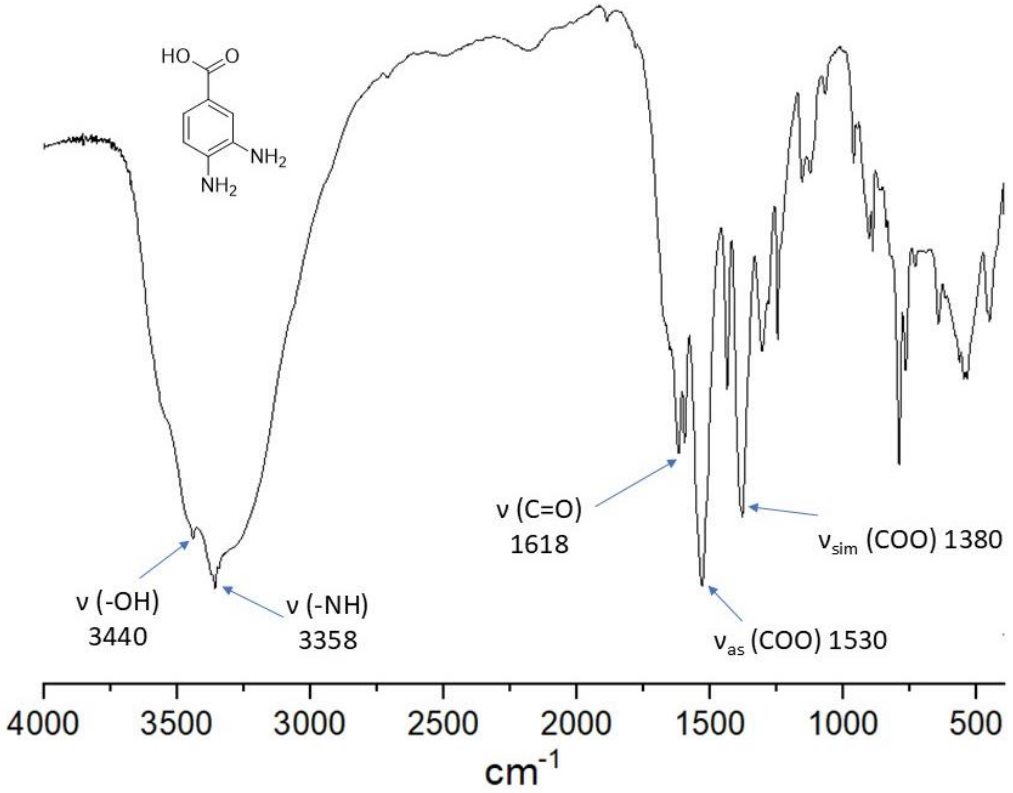

Figure S2. FTIR spectrum of 3,4-diaminobenzoic acid (DABA).

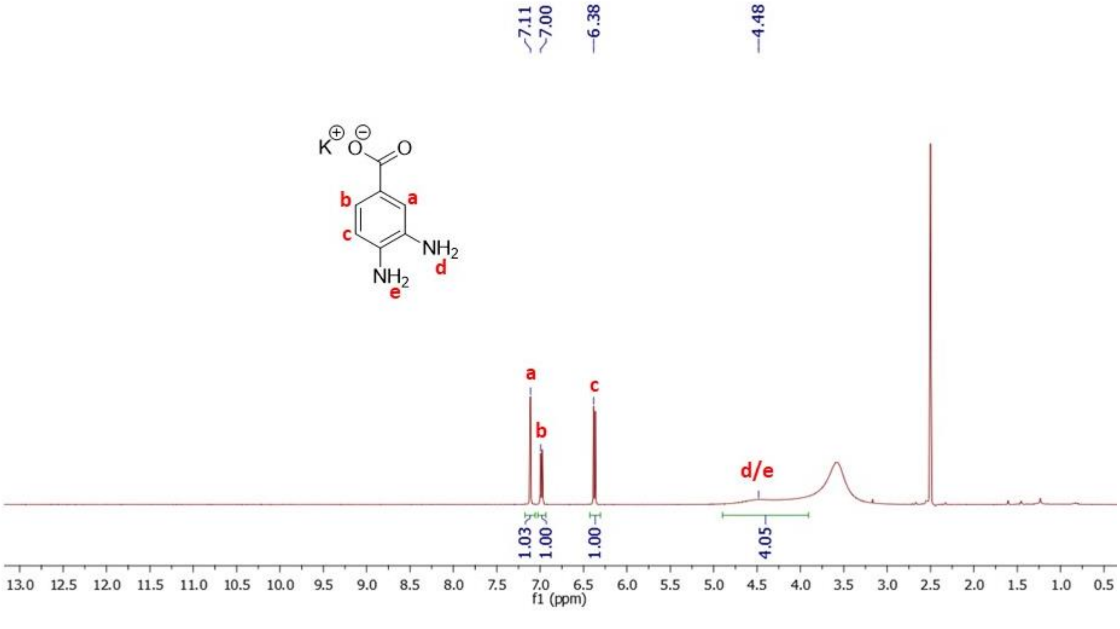

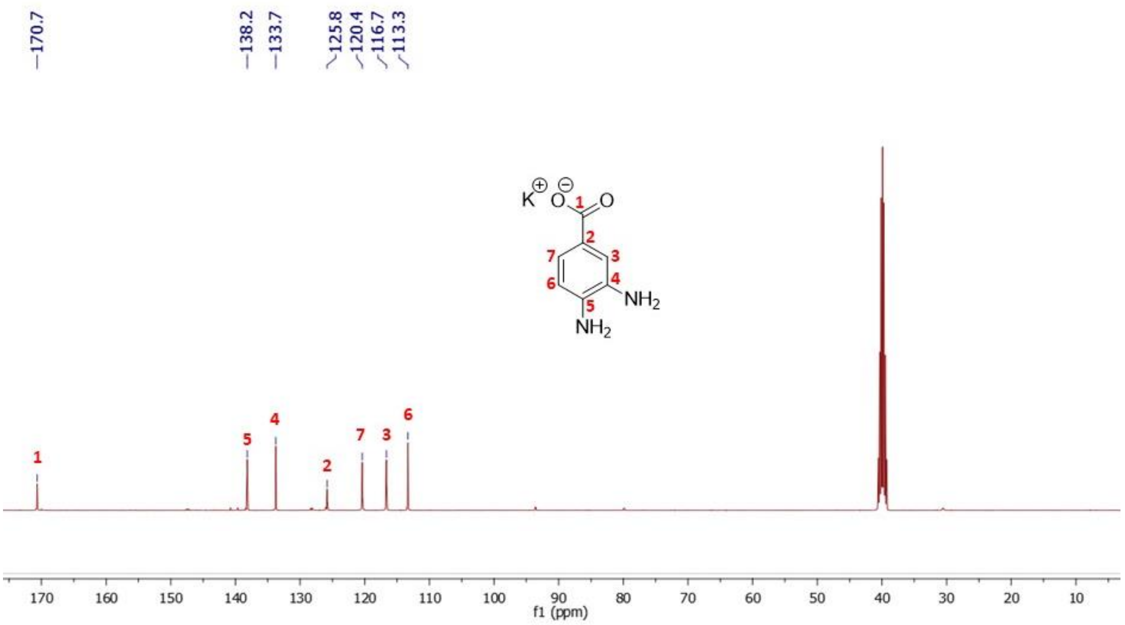

Figure S3. ^{1}H NMR and ^{13}C NMR spectra of potassium 3,4-diaminobenzoate (KDAB) in DMSO-d_6.

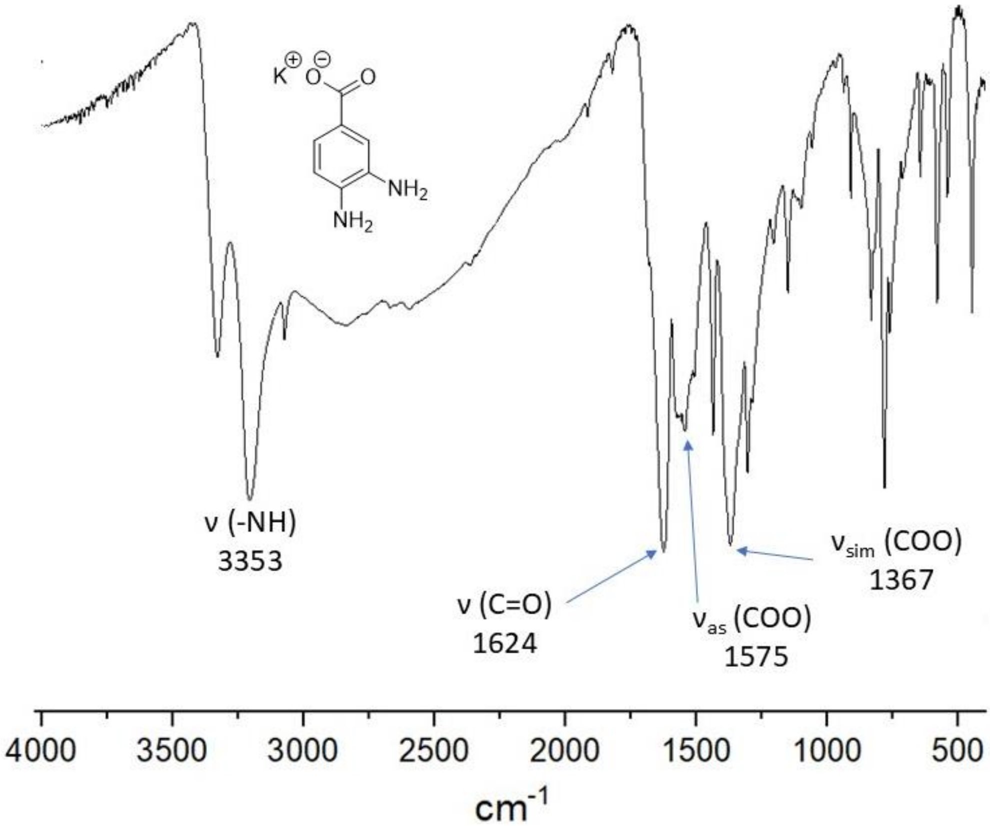

Figure S4. FTIR spectrum of potassium 3,4-diaminobenzoate (KDAB).

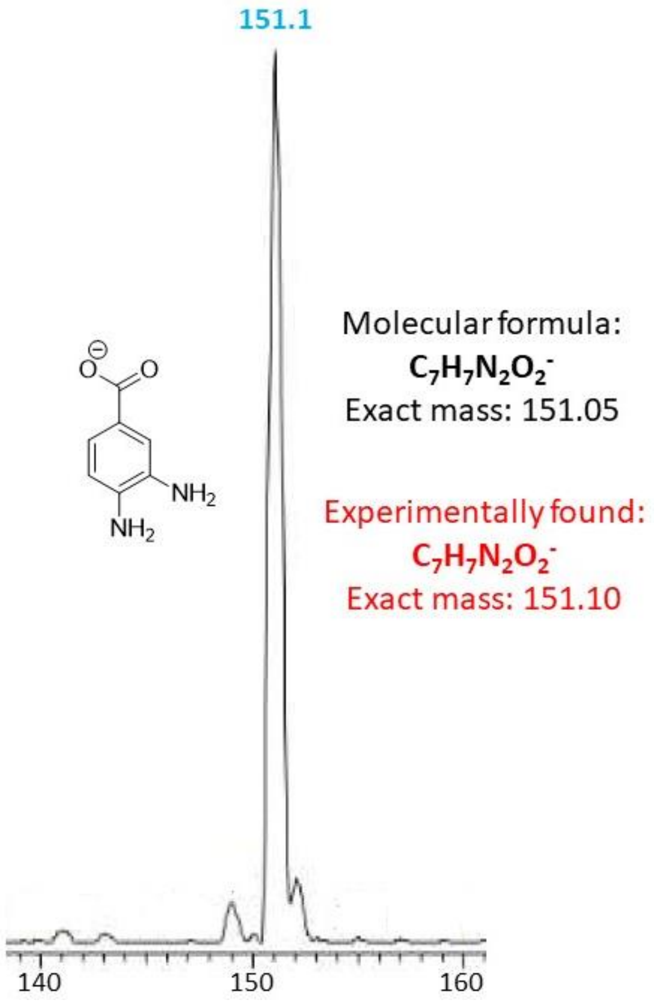

Figure S5. Expanded region of the HRMS ($HESI^-$) spectrum of potassium 3,4-diaminobenzoate (KDAB).

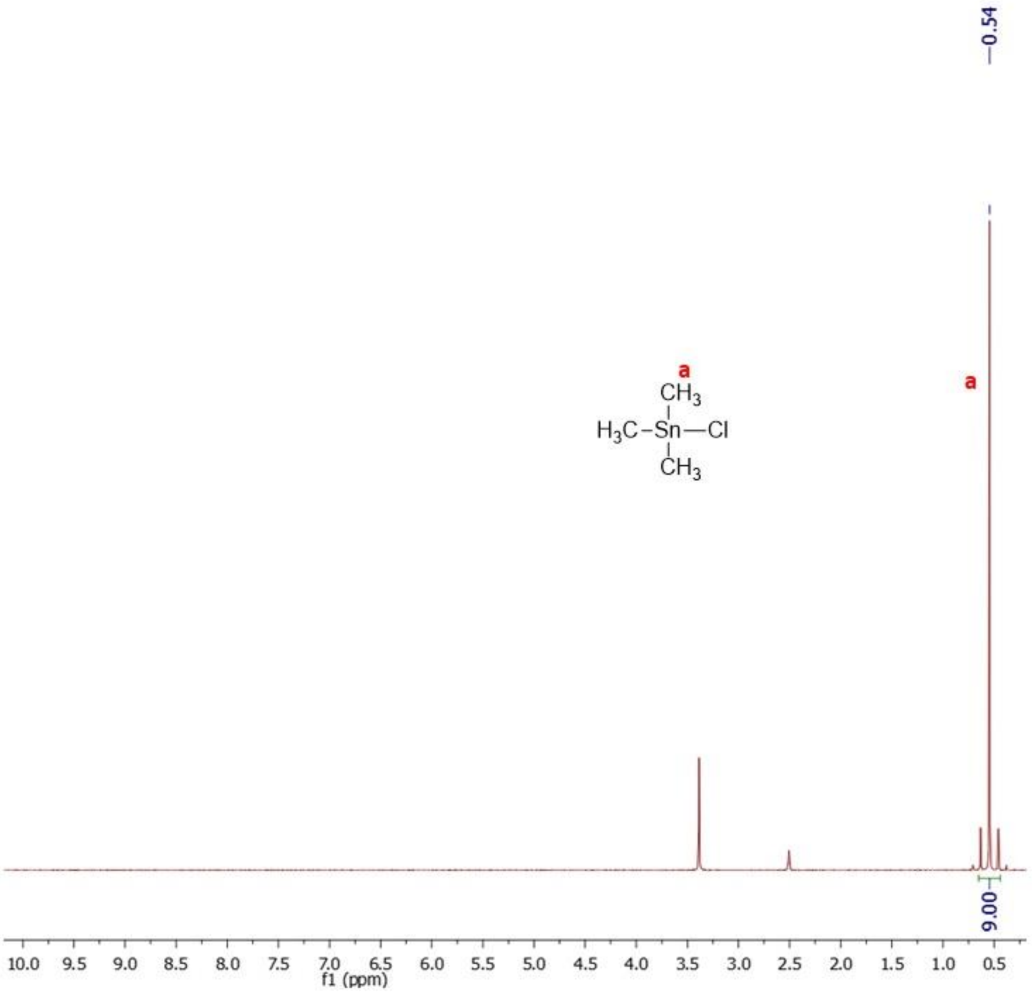

Figure S6. ^{1}H NMR spectrum of the starting compound $SnMe_3Cl$ in DMSO-d_6.

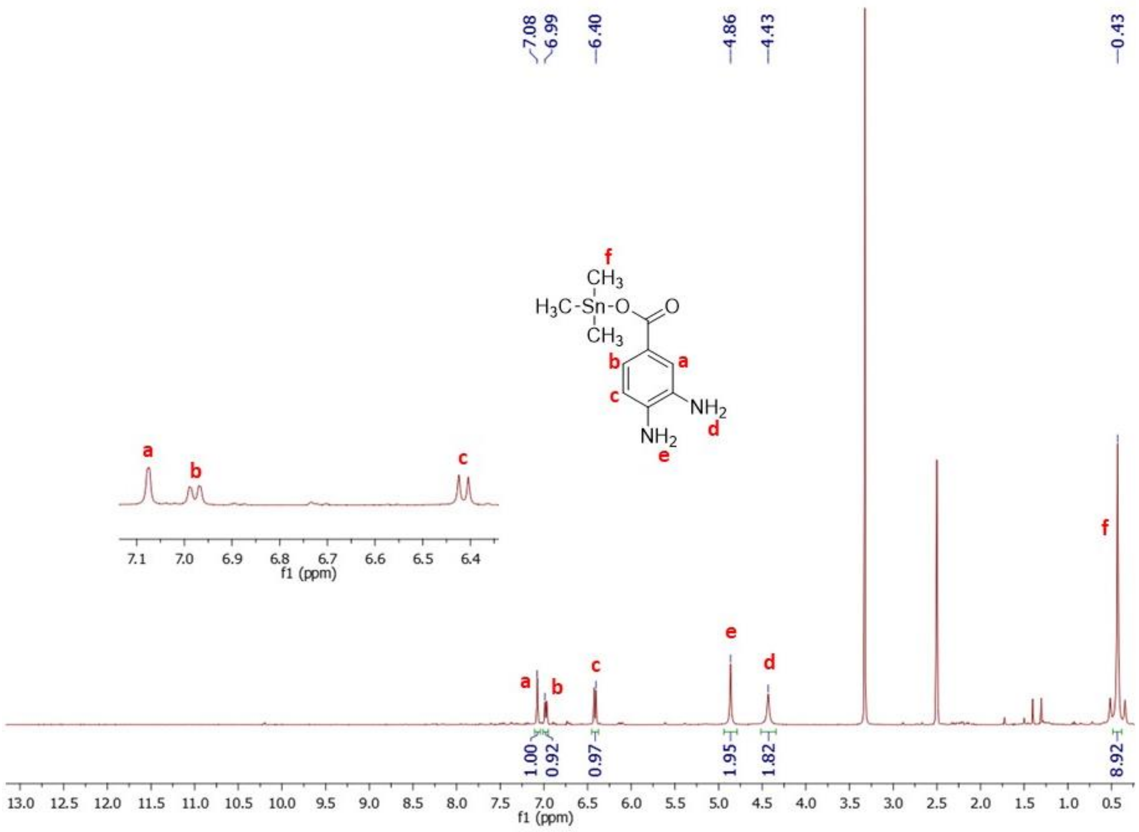

7.08
6.99
6.40
4.86
4.43
0.43
f
CH_3
H_3C-Sn-O
O
CH_3
b
a
c
NH_2
d
NH_2
e
a
b
c
7.1 7.0 6.9 6.8 6.7 6.6 6.5 6.4
f1 (ppm)
f
e
c
d
a
b
1.00
0.92
0.97
1.95
1.82
8.92
13.0 12.5 12.0 11.5 11.0 10.5 10.0 9.5 9.0 8.5 8.0 7.5 7.0 6.5 6.0 5.5 5.0 4.5 4.0 3.5 3.0 2.5 2.0 1.5 1.0 0.5
f1 (ppm)

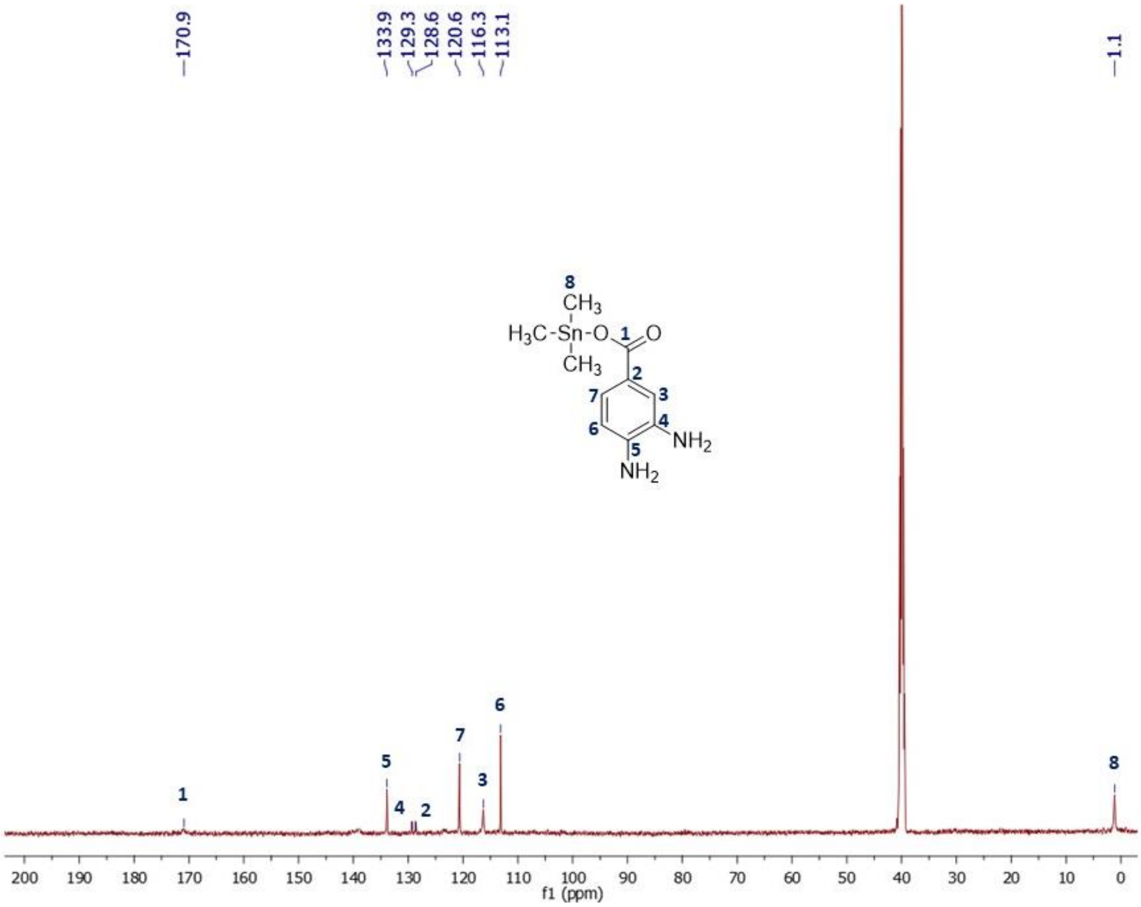

Figure S7. ^{1}H NMR and ^{13}C NMR spectra of Sn(IV)-1 in DMSO-d_6.

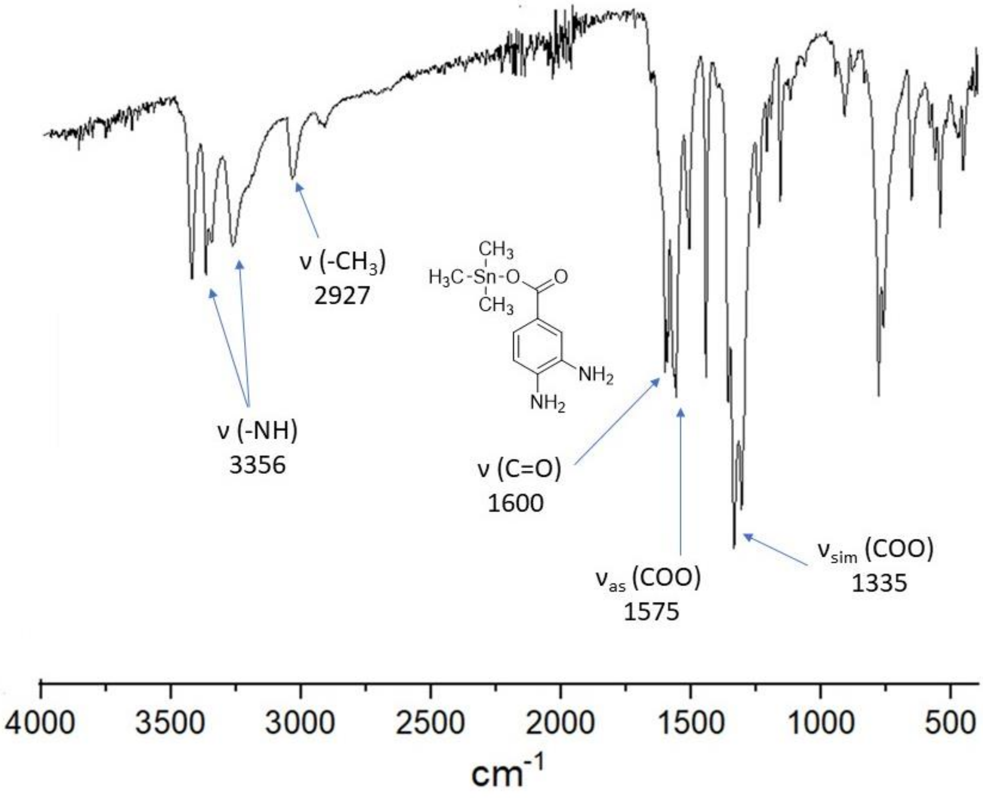

Figure S8. FTIR spectrum of Sn(IV)-1.

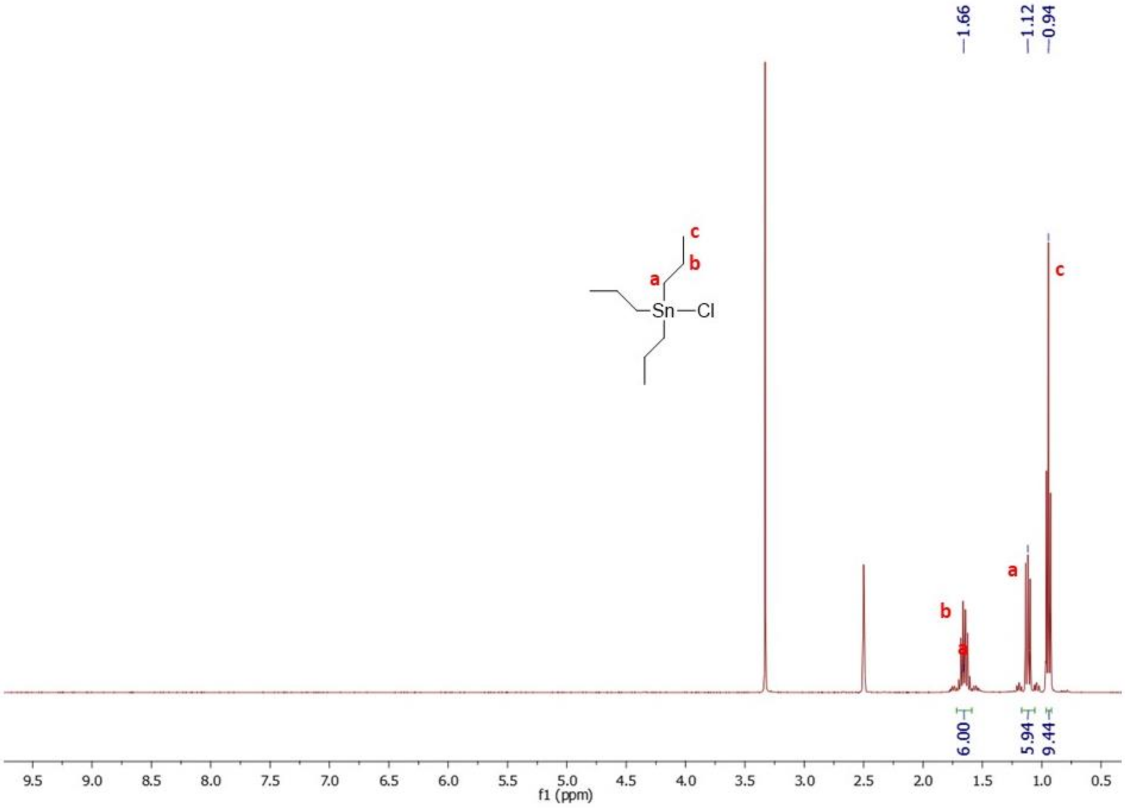

Figure S9. ^{1}H NMR spectrum of the starting complex Sn-(*n*-Pr)$_3$Cl in DMSO-d_6

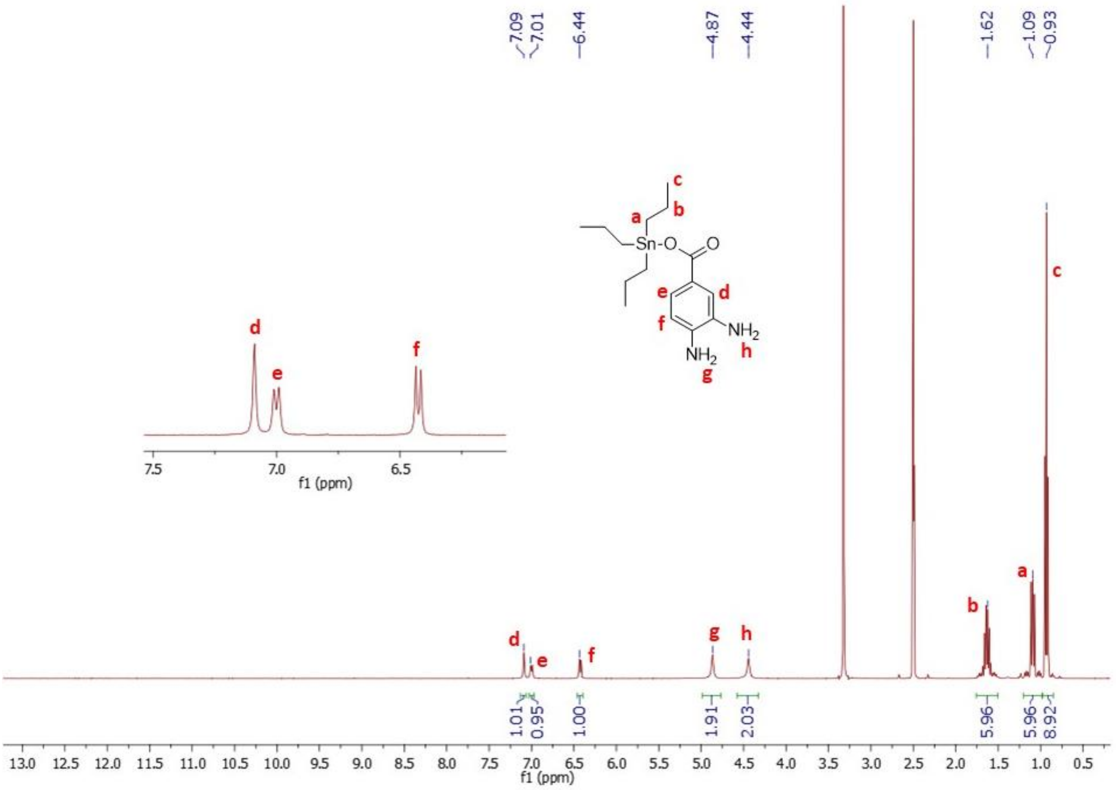

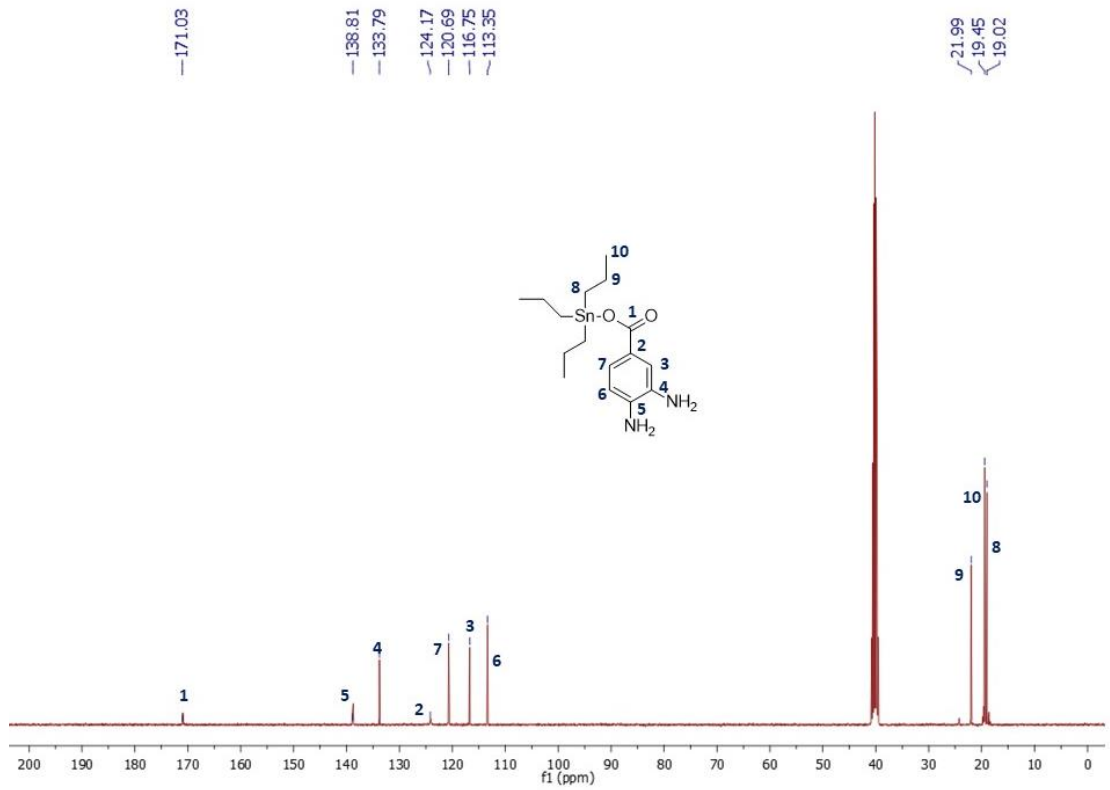

Figure S10. ^{1}H NMR and ^{13}C NMR spectra of Sn(IV)-2 in DMSO-d_6.

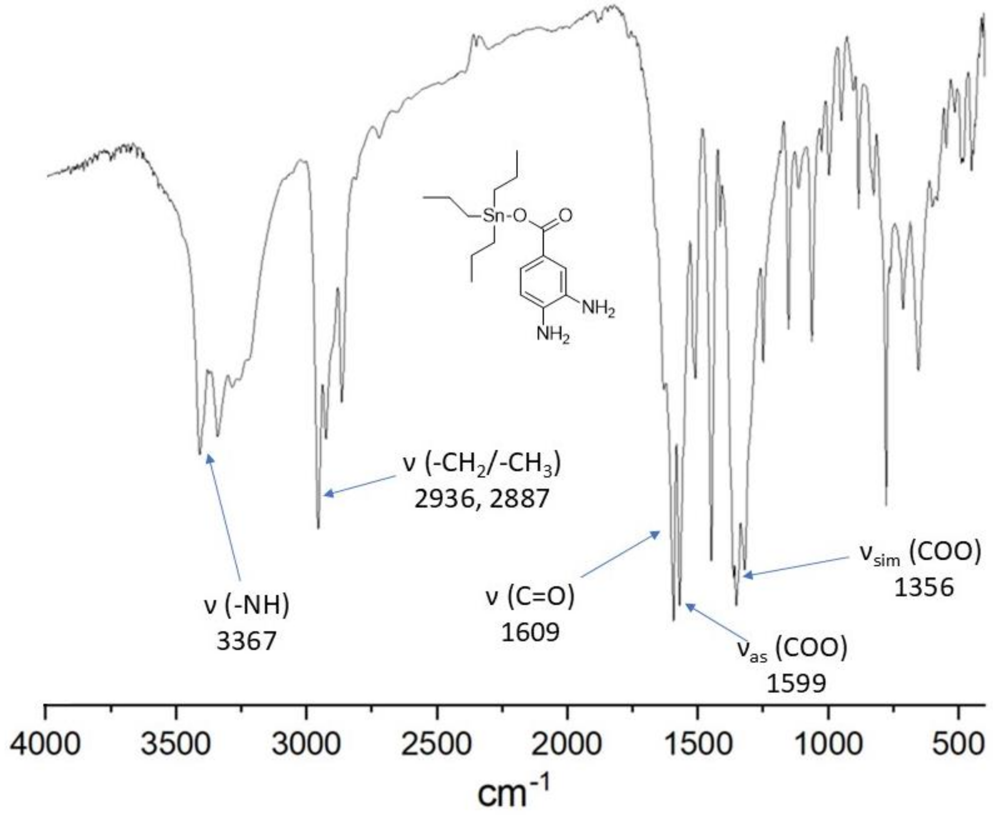

Figure S11. FTIR spectrum of Sn(IV)-2.

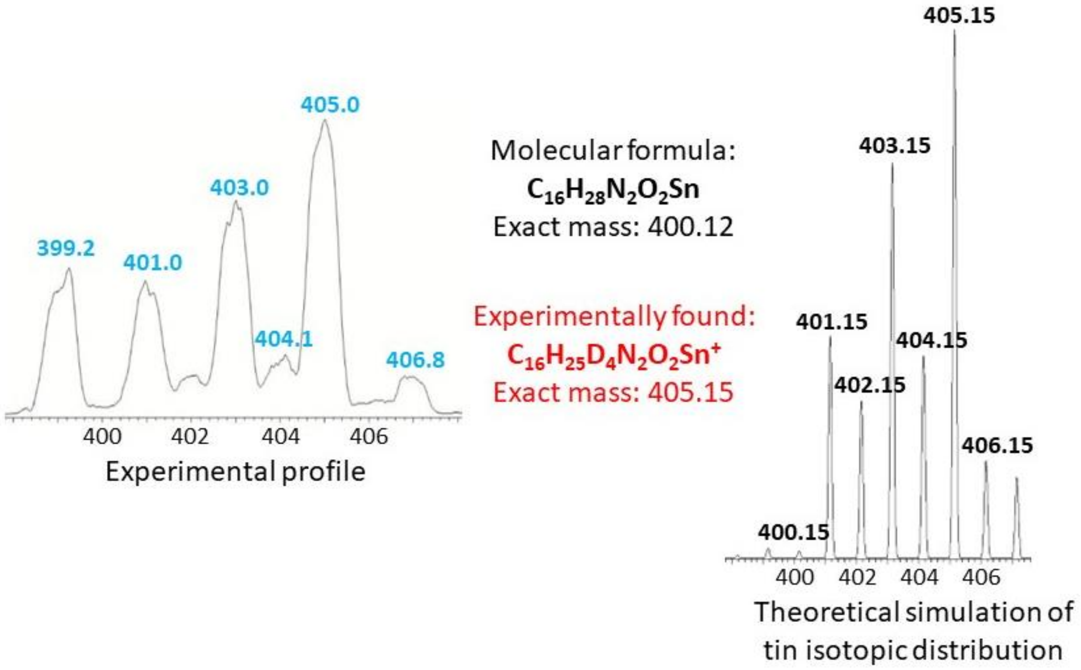

Figure S12. Expanded region of the HRMS (HESI$^+$) spectrum of Sn(IV)-2. Comparison between the experimental isotopic pattern and the theoretical isotopic distribution calculated for the assigned tin-containing ion.

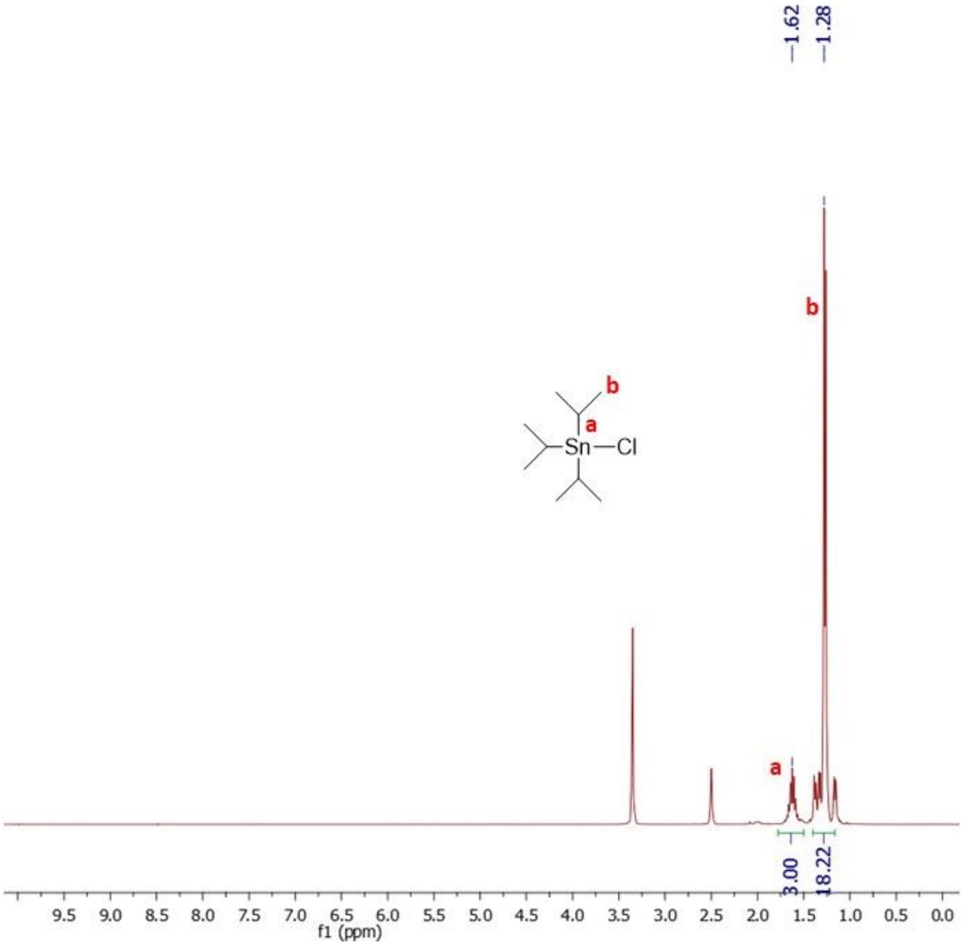

Figure S13. ^{1}H NMR spectrum of the starting complex Sn-(^{i}Pr)$_3$Cl in DMSO-d_6.

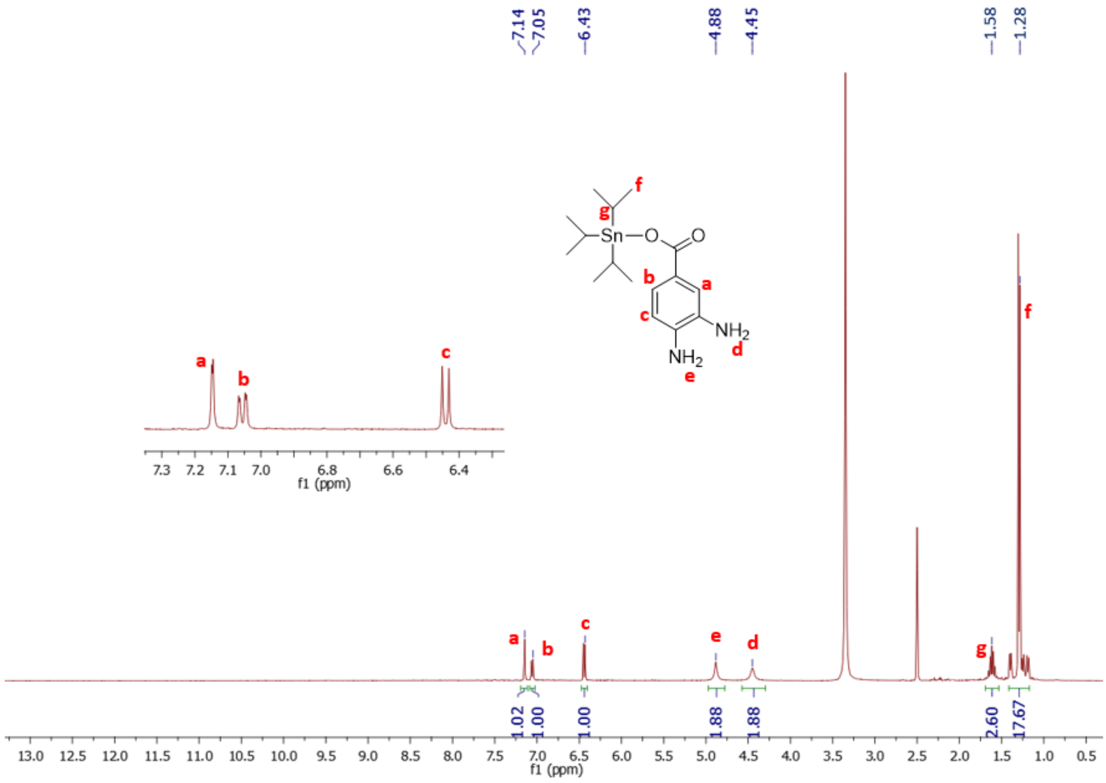

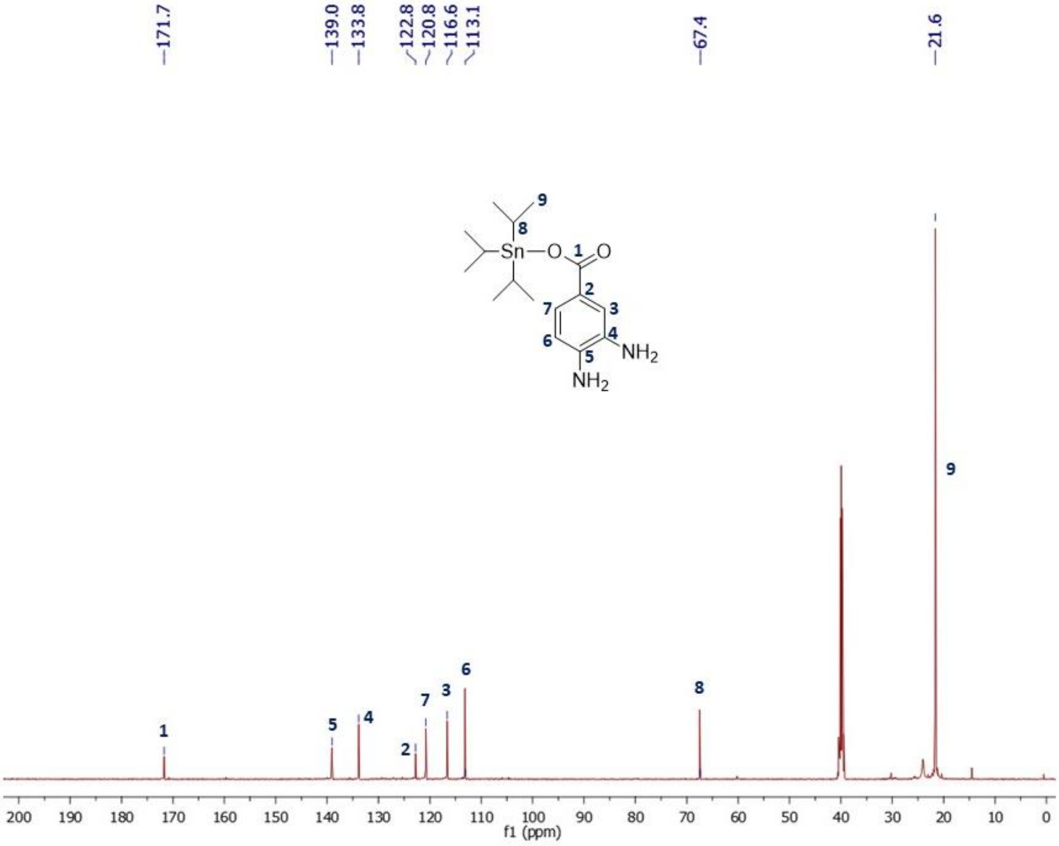

Figure S14. ^{1}H NMR and ^{13}C NMR spectra of Sn(IV)-3 in DMSO-*d6*.

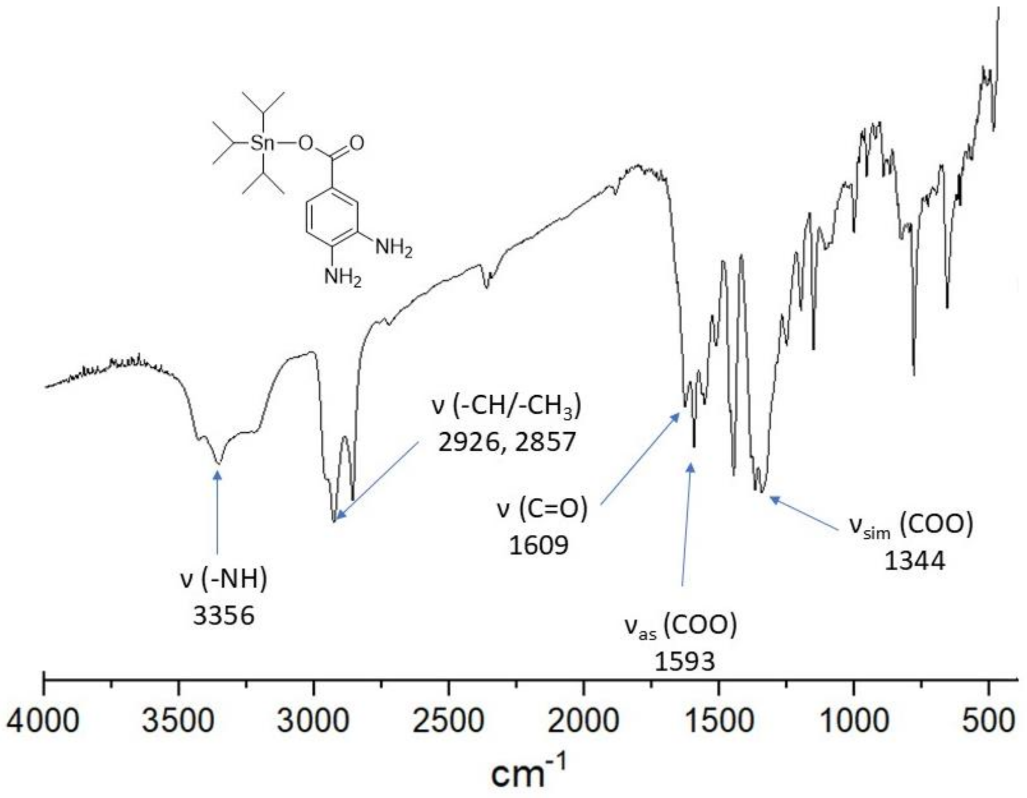

Figure S15. FTIR spectrum of Sn(IV)-3.

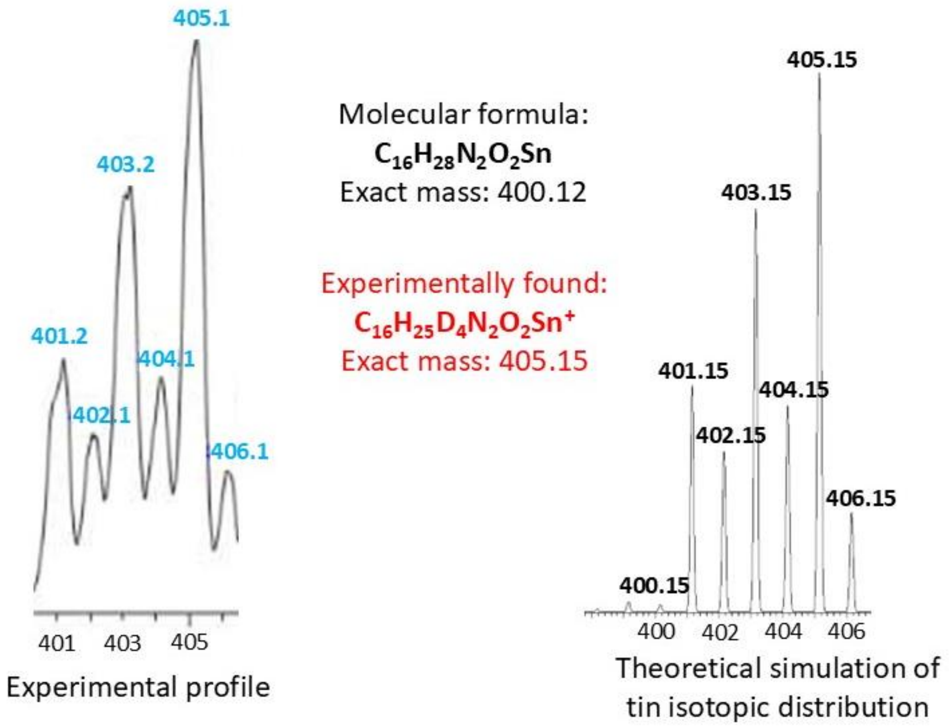

Figure S16. Expanded region of the HRMS (HESI$^+$) spectrum of Sn(IV)-3. Comparison between the experimental isotopic pattern and the theoretical isotopic distribution calculated for the assigned tin-containing ion.

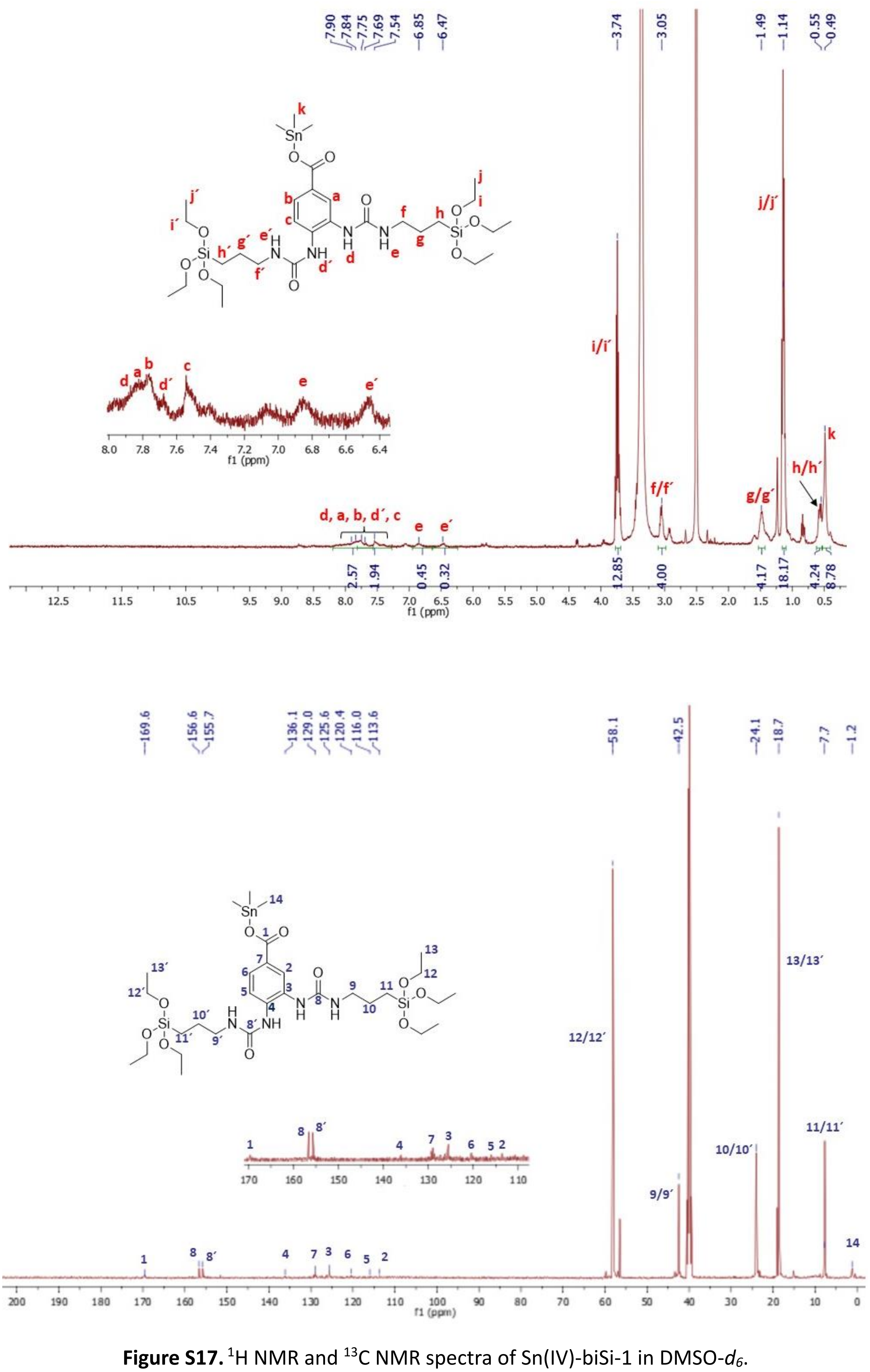

Figure S17. ^{1}H NMR and ^{13}C NMR spectra of Sn(IV)-biSi-1 in DMSO-d_6.

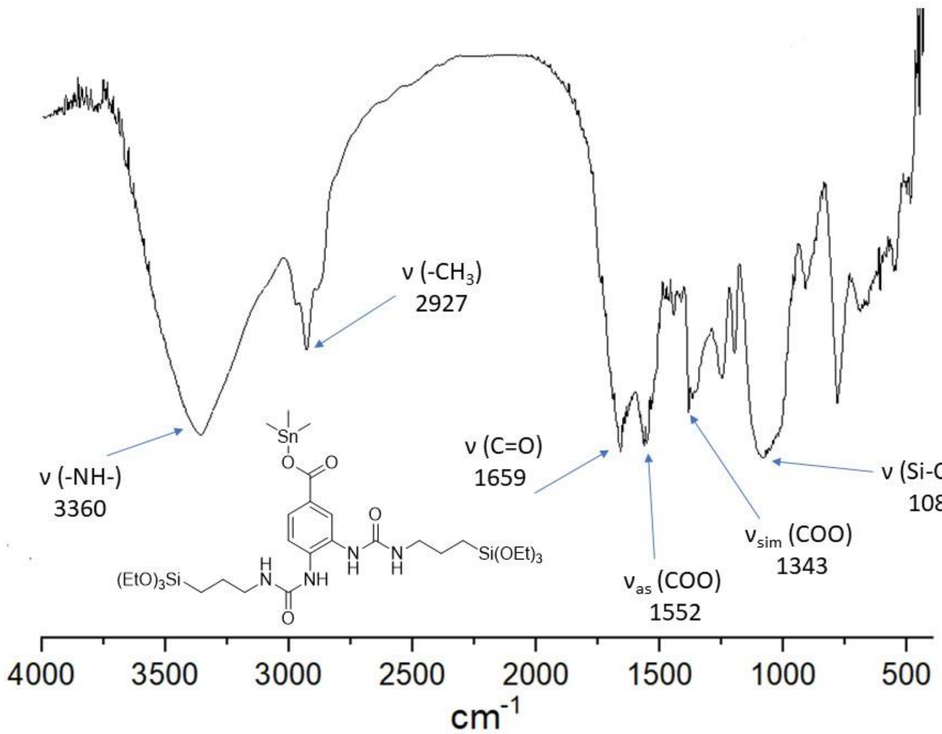

Figure S18. FTIR spectrum of Sn(IV)-biSi-1.

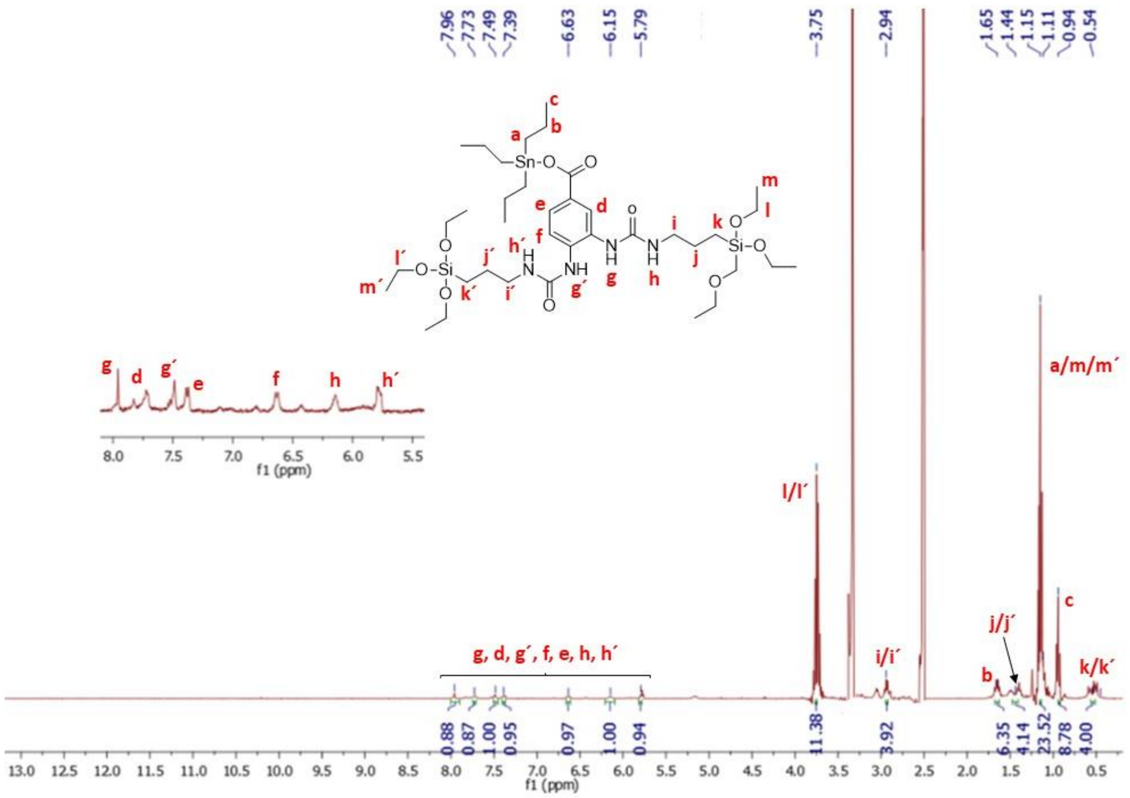

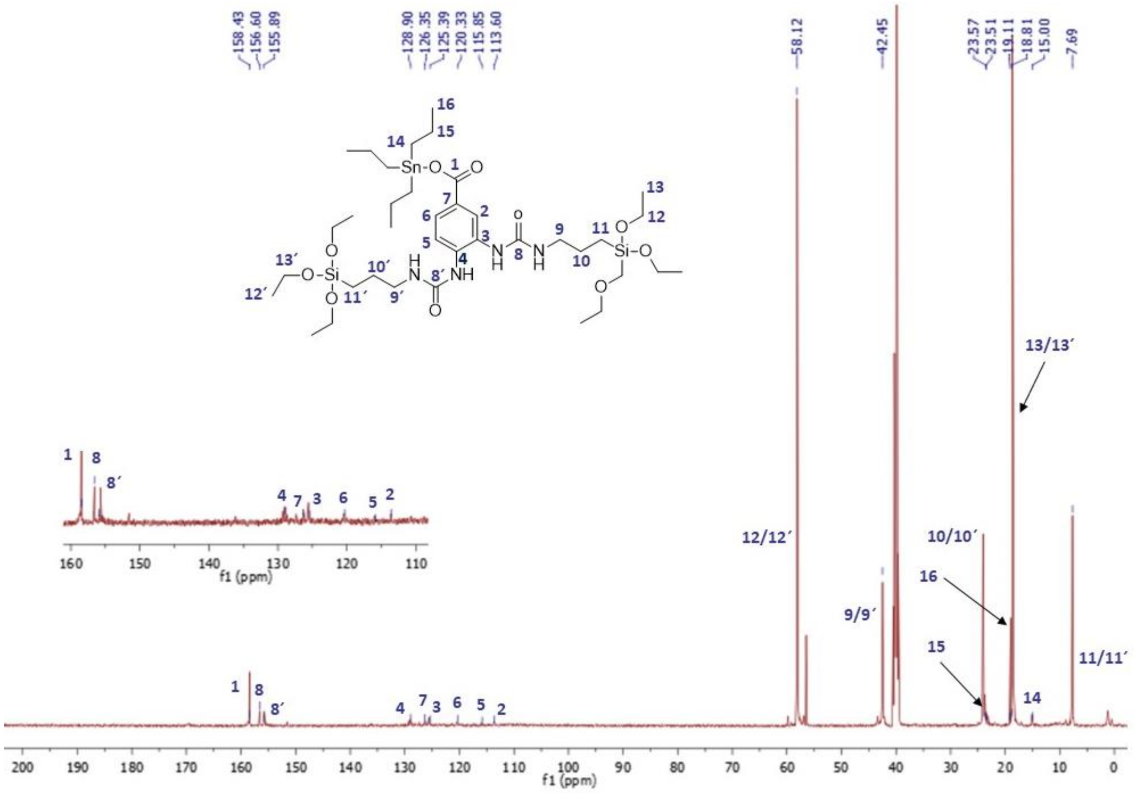

Figure S19. ^{1}H NMR and ^{13}C NMR spectra of Sn(IV)-biSi-2 in DMSO-d_6.

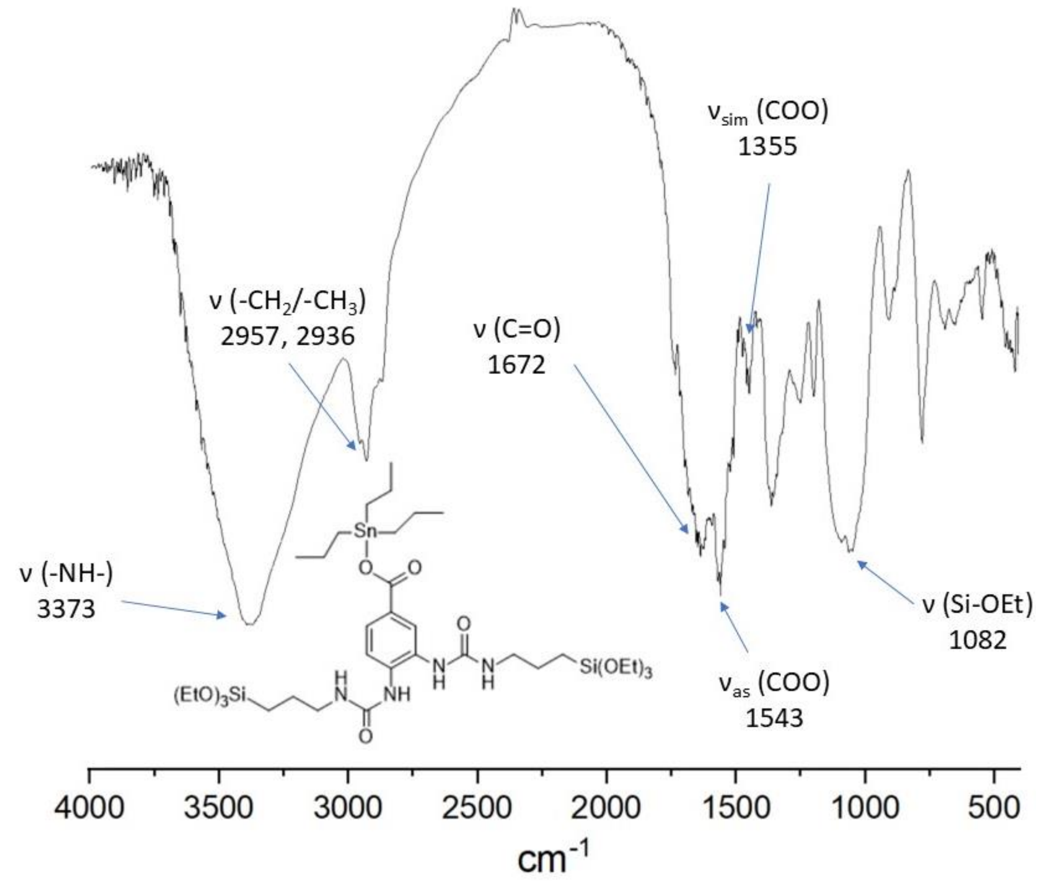

Figure S20. FTIR spectrum of Sn(IV)-biSi-2.

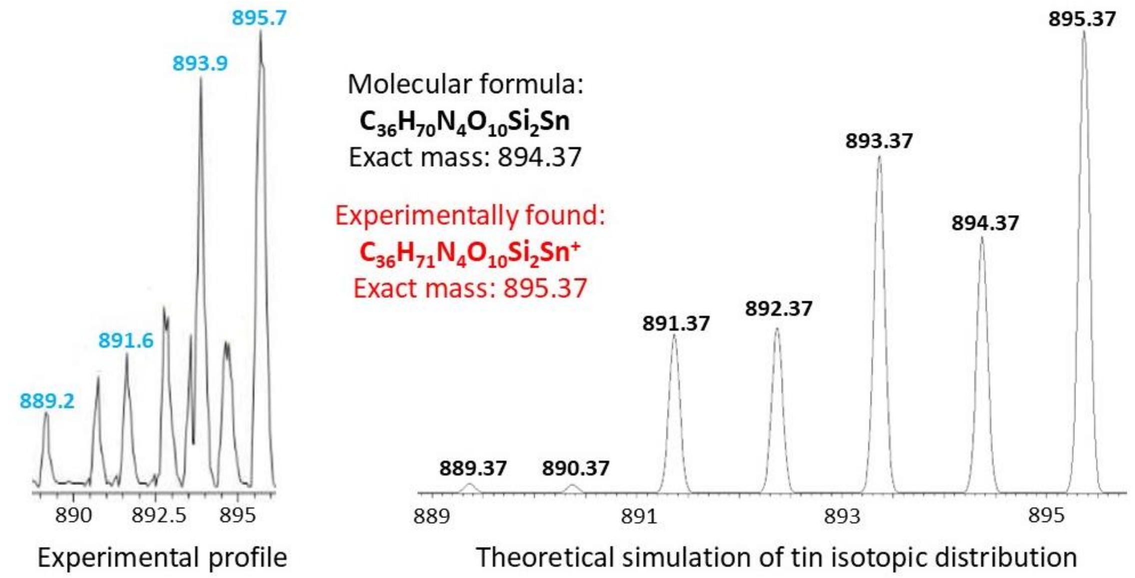

Figure S21. Expanded region of the HRMS (HESI$^+$) spectrum of Sn(IV)-biSi-2. Comparison between the experimental isotopic pattern and the theoretical isotopic distribution calculated for the assigned tin-containing ion.

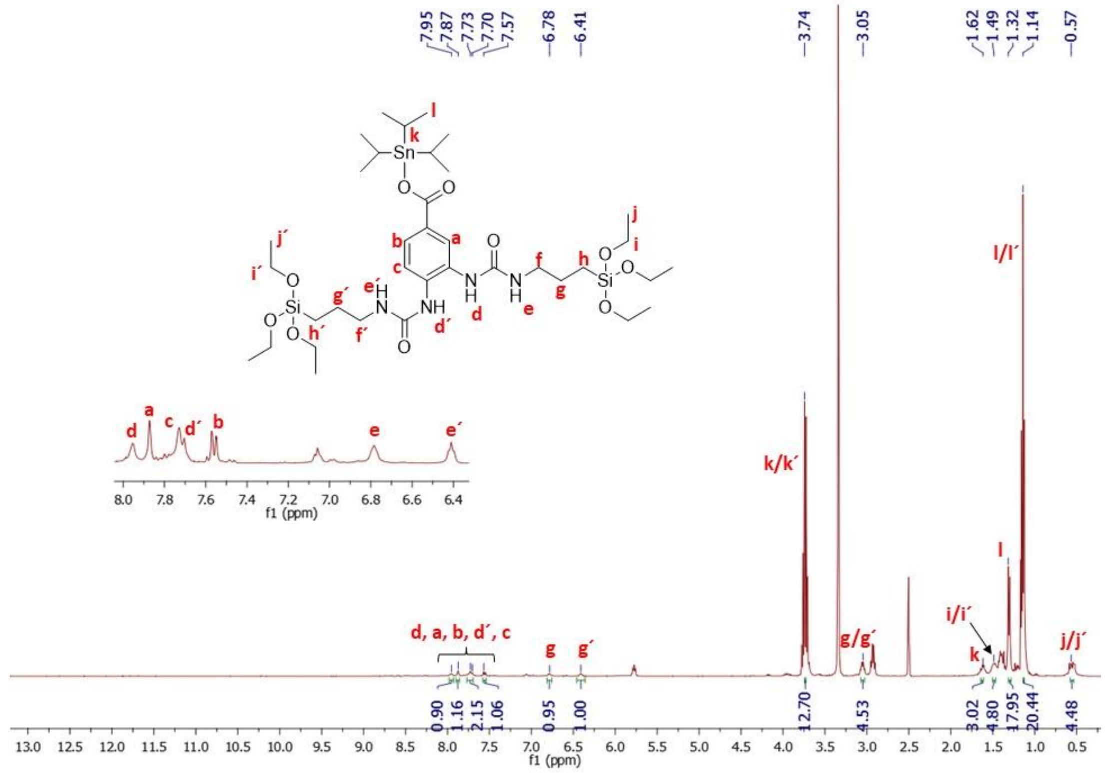

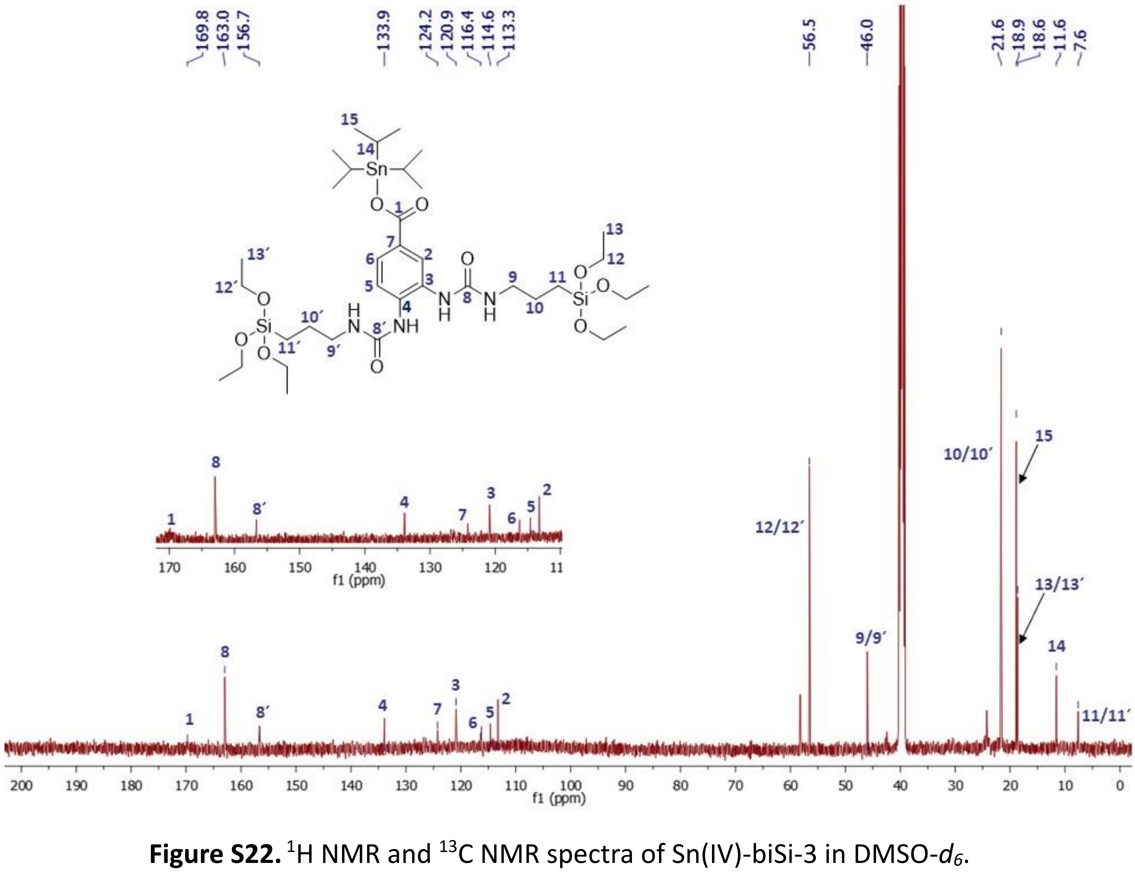

Figure S22. ^{1}H NMR and ^{13}C NMR spectra of Sn(IV)-biSi-3 in DMSO-d_6.

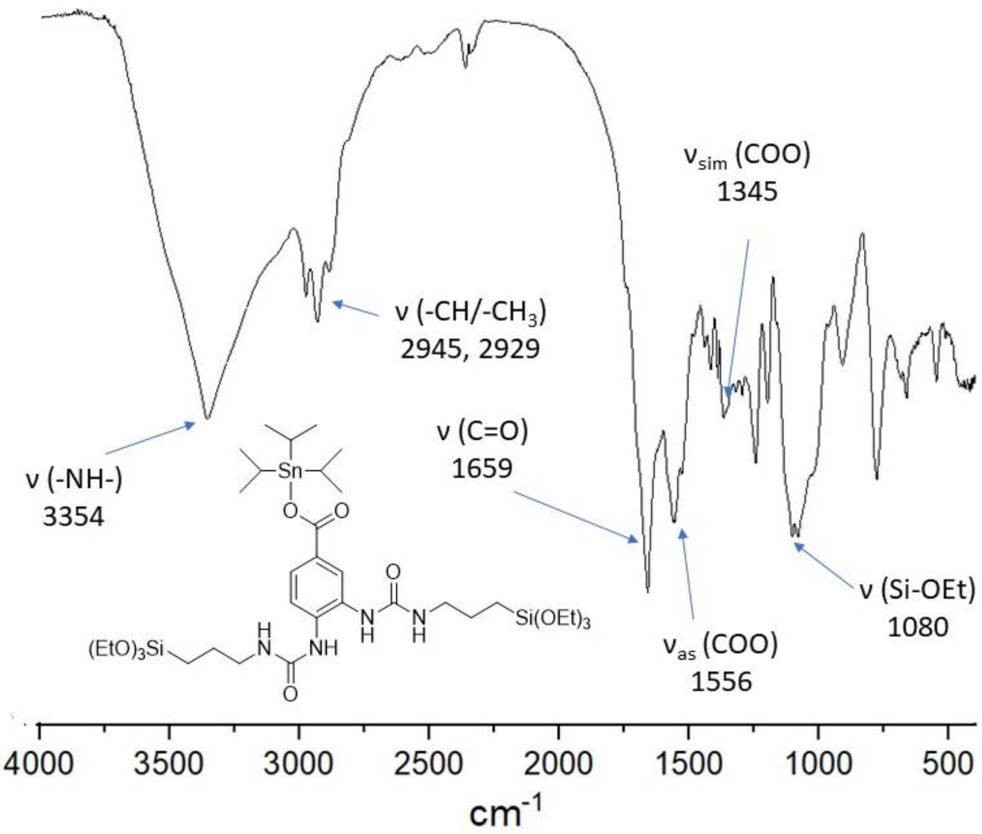

Figure S23. FTIR spectrum of Sn(IV)-biSi-3.

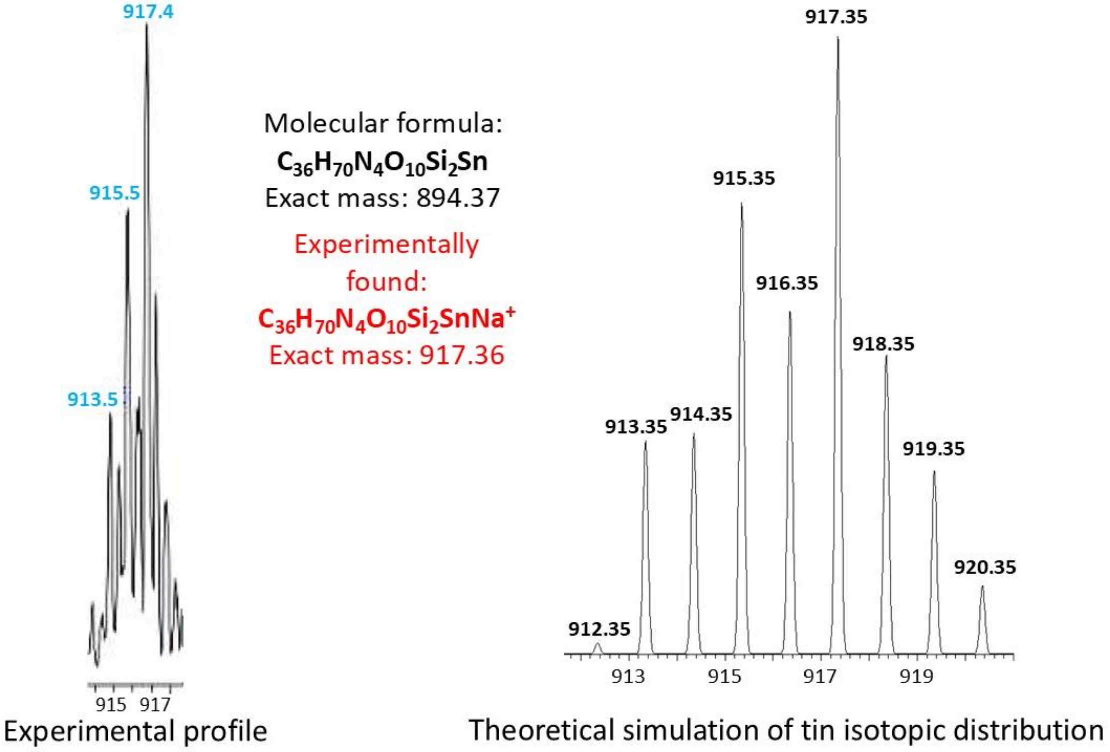

Figure S24. ed region of the HRMS (HESI⁺) spectrum of Sn(IV)-biSi-3. Comparison between the experimental isotopic pattern and the theoretical isotopic distribution calculated for the assigned tin-containing ion.

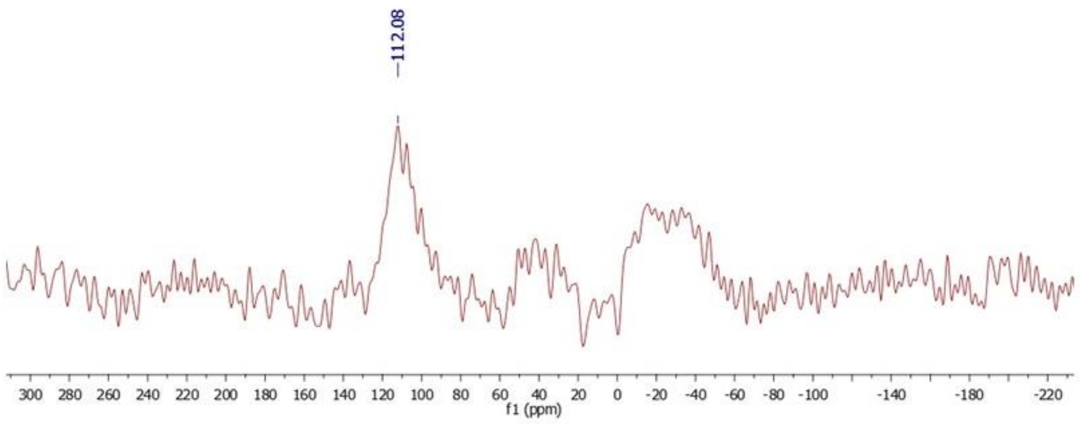

Figure S25. Solid-state CP ^{119}Sn NMR spectrum of Sn(IV)-biSi-1.

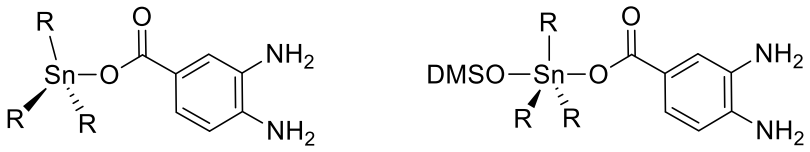

Scheme S1. Geometries adopted by the Sn(IV) precursors in solution depending on the solvent used. Left) quasi-tetrahedral geometry using the non-coordinating solvent chloroform. Right) trigonal bipyramidal geometry with the DMSO directly coordinated to the tin(IV) ion.
